# Hypoxia Selectively Increases a SMAD3 Signaling Axis to Promote Cancer Cell Invasion

**DOI:** 10.3390/cancers14112751

**Published:** 2022-06-01

**Authors:** Karine Brochu-Gaudreau, Martine Charbonneau, Kelly Harper, Claire M. Dubois

**Affiliations:** Department of Immunology and Cell Biology, Université de Sherbrooke, Sherbrooke, QC J1H 5N4, Canada; karine.brochu-gaudreau@usherbrooke.ca (K.B.-G.); martine.charbonneau@usherbrooke.ca (M.C.); kelly.harper@usherbrooke.ca (K.H.)

**Keywords:** transforming growth factor β, invadopodia, metastasis, chorioallantoic membrane xenograft assay, SMAD3 signaling

## Abstract

**Simple Summary:**

While 90% of cancer patient deaths are caused by cancer dissemination through metastasis, no current therapy selectively targets this process due to the lack of selective inhibitors. Various intratumoral factors are known to promote metastasis, notably low oxygen concentration, called hypoxia, and various growth factors, including transforming growth factor β (TGFβ). Our group previously demonstrated that hypoxia enhances TGFβ-induced cancer cell invasion processes. The present study further characterizes the mechanisms involved in hypoxia-induced cancer cell invasion using in vitro, in vivo and in silico approaches and identifies a HDAC6- SMAD3 pathway that can be pharmacologically targeted to inhibit tumor progression.

**Abstract:**

Transforming growth factor β (TGFβ) plays a paradoxical role in cancer, first inhibiting then promoting its progression, a duality that poses a real challenge for the development of effective TGFβ-targeted therapies. The major TGFβ downstream effectors, SMAD2 and SMAD3, display both distinct and overlapping functions and accumulating evidence suggests that their activation ratio may contribute to the dual effect of TGFβ. However, the mechanisms responsible for their selective activation remain poorly understood. Here, we provide experimental evidence that hypoxia induces the pro-invasive arm of TGFβ signaling through a selective increase in SMAD3 interaction with SMAD-Anchor for Receptor Activation (SARA). This event relies on HDAC6-dependent SMAD3 bioavailability, as well as increased SARA recruitment to EEA1+ endosomes. A motility gene expression study indicated that SMAD3 selectively increased the expression of *ITGB2* and *VIM*, two genes that were found to be implicated in hypoxia-induced cell invasion and associated with tumor progression and metastasis in cohorts of cancer patients. Furthermore, CAM xenograft assays show the significant benefit of selective inhibition of the SMAD3 signaling pathway as opposed to global TGFβ inhibition in preventing tumor progression. Overall, these results suggest that fine-tuning of the pro-invasive HDAC6-SARA-SMAD3 axis could be a better strategy towards effective cancer treatments.

## 1. Introduction

Transforming growth factor β (TGFβ) is a ubiquitously expressed growth factor involved in a variety of normal and pathological cellular processes, ranging from cell differentiation and apoptosis to cell invasion and tumor immune evasion (reviewed in [1,2]). TGFβ is frequently overexpressed in solid tumors [3,4,5,6], and its expression is correlated with tumor progression and metastasis in human cancers [7,8,9]. Due to its central role in tumor progression, many compounds targeting TGFβ signaling have been developed for therapeutic purposes [10]. Disappointingly, most of these molecules have shown limited or no activity in clinical trials [11,12]. This was presumably due to the concomitant inhibition of both normal and pathological TGFβ signaling activities. TGFβ is indeed known to play a dual/biphasic role in tumor progression. Initially acting as a tumor suppressor, due to its cytostatic program that induces cell cycle arrest in G1 phase and regulates apoptosis, TGFβ signaling subsequently switches to bypass this protective program and promote various pro-tumorigenic and pro-invasive functions of cancer cells throughout tumor progression [2,13,14].

Canonical TGFβ signaling is initiated by the binding of TGFβ to its receptor, TGFBR2, which allows this receptor to recruit and phosphorylate TGFBR1. The downstream cytosolic effectors, SMAD2 and SMAD3 transcription factors, are then recruited to the activated TGFBR1, which, in turn, phosphorylates SMAD2/3 at their C-terminal SSXS domain. Activated SMAD2/3 (pSMADs) can then oligomerize with SMAD4, allowing the translocation of the SMAD complex to the nucleus and subsequent gene regulation (reviewed in [15]). The importance of TGFβ downstream effectors, SMAD2 and SMAD3, in the pro- and anti-tumoral effects of TGFβ has been demonstrated in vitro and in vivo [16,17,18]. In addition, recent studies suggest the use of SMAD2/3 expression or activation level as a prognostic marker in various cancers [19,20,21]. Despite their 84% protein sequence homology and similar mode of activation, increasing evidence indicates that SMAD2 and SMAD3 perform distinct and opposing functions during tumor progression, including cell invasion, tumor growth, and metastasis [18,22,23], suggesting that SMAD2/SMAD3 expression or activation ratio could be determining factors in the pro- or anti-tumorigenic roles of TGFβ.

The intracellular level of pSMAD2/3 is known to be controlled by multiple cellular processes ranging from SMAD expression to nucleocytoplasmic trafficking [15]. SMAD2/3 are ubiquitously expressed, but studies have shown that their expression can be modulated by different stimuli present in the tumor microenvironment [24,25]. In addition, SMAD2/3 can be sequestered by binding to the microtubule network [26] or the transmembrane prostate androgen-induced protein (TMEPAI) [27], preventing the recruitment of SMAD2/3 to the activated TGFBR and subsequent effects on gene regulation. However, the mechanisms responsible for the selective activation of SMAD2 versus SMAD3 during cancer progression are still poorly understood. 

Tumor hypoxia is among the stimuli known to influence TGFβ signaling. Hypoxia is a fundamentally important feature of solid tumors known to drive cancer progression and metastasis and to correlate with poor prognosis in a wide variety of cancers, including pancreatic, cervical, and brain [28,29,30,31]. Hypoxia has been described to increase the expression [32] and bioavailability [33,34] of TGFβ. In addition, hypoxia has been shown to induce SMAD2 proteolysis [35] and to stimulate SMAD3 transcriptional activity [36]. We previously demonstrated that hypoxia selectively increases the phosphorylation of SMAD3 (pSMAD3) in the HT-1080 cancer cell line, a process that was required for the hypoxia-induced increase in cancer cell invasion [37], suggesting that hypoxia induces a pSMAD equilibrium bias that might contribute to the shift in TGFβ signaling from the anti-tumorigenic to the pro-tumorigenic state.

In this study, we sought to determine the mechanism by which hypoxia regulates SMAD3 signaling in cancer and how this affects cancer progression and metastasis. We demonstrate that the selective activation of SMAD3 signaling in hypoxia occurs in a wide variety of cancers and that SMAD2 and SMAD3 exert dichotomous roles in cell invasion, in which SMAD3 drives the invasive properties of cancer cells in vitro and in vivo. The identified mechanism involves a hypoxia-induced HDAC6-SARA-SMAD3- axis that contributes to the ability of cancer cells to degrade the extracellular matrix and form metastases. It also involves ITGB2 and VIM as potential downstream targets of SMAD3 action.

## 2. Materials and Methods

### 2.1. Plasmid DNA

Mission shRNA plasmids targeting *SMAD2* (TRCN0000010477, TRCN0000010478), *SMAD3* (TRCN000033055, TRCN0000330128), *RHOB* (TRCN0000047848, TRCN0000047849), *ITGB2* (TRCN0000029643, TRCN0000236135), *VIM* (TRCN0000029120, TRCN0000029121), or scrambled control were from Sigma-Aldrich (Oakville, ON, Canada). SMAD overexpression plasmids, pCMV5b-SMAD2 and pCMV5b-SMAD3, as well as their mutants incapable of C-terminal phosphorylation, pCMV5b-SMAD2-3SA and pCMV5b-SMAD3-3SA [38], were kindly provided by Pr. Attisano (University of Toronto, Canada). VIM-DDK and ITGB2-DDK overexpression plasmids were from Origene (Rockville, MD, USA).

### 2.2. Antibodies and Reagents 

TGFβ1 was a kind gift from Dr F.W. Ruscetti (Biological Response Modifiers Program, FRCF, NCI, Maryland, USA). All fluorophore-coupled reagents (Alexa546-phalloïdin, Alexa488 anti-mouse IgG) were purchased from Invitrogen (Carlsbad, CA, USA). HDAC6 inhibitors (tubacin and CAY10603), SMAD3 inhibitor SIS3 and TGFBR inhibitor Galunisertib (LY2157299) were from Selleckchem (Houston, TX, USA). Cell nucleus stain 4′,6′-diamidino-2-phenylindole (DAPI) was from Invitrogen (Carlsbad, CA, USA). Anti-SMAD2, -SMAD3 and phosphoSMAD2(Ser465/467) and all HRP-coupled secondary antibodies were from Cell Signaling Technology (Boston, MA, USA). Anti-phosphoSMAD3(Ser423/425) was from Abcam (Cambridge, MA, USA). Anti-tubulin and anti-acetylated tubulin were from Sigma Aldrich (Oakville, ON, CA). Anti-SARA and anti-EEA1 antibodies were from Santa Cruz Biotechnology (Santa Cruz, CA, USA). Rabbit TrueBlot was purchased from Rockland Immunochemicals (Limerick, PA, USA). Pimonidazole hydrochloride and anti-pimonidazole mouse antibody were purchased from Hypoxyprobe (Burlington, MA). ITGB2 blocking antibody was from Novus Biologicals (Centennial, CO, USA). Anti-DDK antibody was from Origene. 

### 2.3. Cell Culture and Treatment

Human fibrosarcoma HT-1080, lung adenocarcinoma A549; and invasive breast carcinoma MDA-MB-231 cell lines (ATCC, Manassas, VA, USA) were cultured in Eagle’s minimal essential medium (EMEM; Wisent, Saint-Bruno QC, Canada) at 37 °C in a 5% CO_2_ humidified incubator. Each medium was supplemented with 10% heat inactivated fetal bovine serum (Invitrogen, Carlsbad, CA, USA) and 40 mg/L of gentamicin sulfate (Wisent, Saint-Bruno QC, Canada). All cells were routinely tested to be free of mycoplasma contamination by DAPI staining. Hypoxic conditions were used as follows: cells were serum-starved then placed in a sealed humidified chamber (In VivO_2_ 400 hypoxic workstation; Ruskinn, Bridgend, UK) maintained at 1% O_2_, 5% CO_2_ and balanced in N_2_ for different periods of time, as indicated in the figure legends. For experiments using TGFβ1 and/or pharmacological inhibitors, cells were pre-treated for 30 min with the inhibitor before the addition of TGFβ1.

### 2.4. Immunofluorescence Staining and Analysis

Twenty thousand cells per well were seeded on a coverslip placed at the bottom of a 12-well plate and allowed to adhere for 24 h. Cells were serum starved for 4 h prior to incubation for 3 h in normoxic (21% O_2_) or hypoxic (1% O_2_) conditions, or in the presence of TGFβ1 (1 ng/mL), as described in the figure legends. Immunofluorescence staining of phospho-SMADs, SARA, and EEA1, and image acquisition was performed as previously described [37]. Images were analyzed using FluoView software and the nuclear accumulation of phosphorylated SMADs (pSMADs) or SARA-EEA1 colocalization was calculated according to the formula: (number of co-localized pixels/total number of positive pixels for control (DAPI or EEA1)) × 100. Data from three independent experiments, comprising images of 10 to 20 cells per experiment, were used for statistical analysis.

### 2.5. Chorioallantoic Membrane (CAM) Xenograft Tumor Assay

White leghorn chicken fertilized eggs were obtained from the Public Health Agency of Canada (Nepean, ON, Canada). All experimental procedures involving embryos were conducted in accordance with regulations of the Canadian Council on Animal Care and the Ethics Committee on Animal Research of the University of Sherbrooke (Protocol #054-13). CAM assays were performed as previously described [39], with the following modifications. When indicated, cell suspensions were mixed with the appropriate inhibitors (SIS3, CAY10630, or LY2157299) or vehicle, prior to grafting onto the CAM. Following grafting, the eggs returned to the incubator for 7 days. Forty-five minutes prior to sample collection, pimonidazole was injected intravenously into the CAM vasculature. Following sacrifice, xenografts were removed and fixed in 4% PFA and submitted to sucrose solution gradient (4–15%) to avoid water crystal formation, before cryo-embedding in OCT (Fisher Scientific, Ottawa, ON, Canada), following the manufacturer’s instructions. Livers from chick embryo were also collected, immediately frozen in liquid nitrogen and cryopreserved at −80 °C.

### 2.6. Xenograft Immunofluorescence and Quantitation

Immunofluorescence staining for pimonidazole was performed as previously described [39] on 5µm thick xenograft cryosections. Tumor sections were double stained for pimonidazole and pSMAD2 or pSMAD3 antibody (1:50). For quantitation of pSMAD staining intensity, a minimum of 6 representative areas from at least 3 different tumors for each condition were captured using an Axioskop 2 phase-contrast/epifluorescence microscope (Carl Zeiss Inc., Oberkochen, Germany). Fluorescence intensities were analyzed using the ImagePro Plus software version 6 (Media Cybernetics, Rockville, MD, USA). The sum of pSMAD labeling intensity (density) relative to the total area of pimonidazole positive or negative areas was calculated.

### 2.7. Genomic DNA Extraction and Alu-Repeats qPCR

Liver tissue was ground in liquid nitrogen and genomic DNA was extracted using DNAzol reagent (Invitrogen), following the manufacturer’s instructions. PCR amplification of primate Alu-repeats was performed as previously described [40] using BiMake SYBR Green MasterMix (BiMake, Houston, TX, USA). A standard curve of human DNA (HT-1080 cells lysate) serially diluted in control chick embryo liver genomic DNA was used to determine the amount of human DNA in liver samples (hepatic metastatic burden). 

### 2.8. Cell Transfection and Transduction

Third generation lentiviral production in HEK293 lenti+ cells (ATCC) was performed by transfection of PLP1, PLP2, PLP-VSVG and pLKO.1 shRNA plasmid using polyehylenimine (PEI) transfection reagent (PolyScience, Niles, IL, USA). After 72 h, the medium containing the produced lentiviral particles was centrifuged 5 min at 1000× *g*, to remove cellular debris, aliquoted and stored at −80 °C until use. HT-1080, MDA-MB-231 and A549 cells were transduced with the lentiviral particles using polybrene (Millipore, Burlington, MA, USA) for 72 h followed by a 0.2 µg/mL puromycin (InvivoGen, San Diego, CA, USA) selection for 5 days. Transient transfection of overexpression plasmids was achieved using TransIT-LT1 reagent (Mirus Bio, Madison, WI, USA), following the manufacturer’s instructions. 

### 2.9. Invadopodia Assay 

Microscopy coverslips were prepared as described [41] using Alexa488-conjugated gelatin (Invitrogen). Thirty thousand cells were seeded per coverslip and incubated under normoxic (21% O_2_), hypoxic (1% O_2_), or TGFβ1-supplemented (1 ng/mL) conditions for 10 h. For experiments involving ITGB2 blocking antibodies, the antibodies were added 30 min before stimulation with TGFβ1 or hypoxia at the concentrations indicated in the figure legends. The cells were then fixed in 2% paraformaldehyde for 15 min, permeabilized and blocked with 0.3% triton in 2% BSA for 30 min. Cell nuclei were stained with DAPI (300 nM) for 5 min at room T° and coverslips were mounted on microscopy slides with Vectashield anti-fade mounting medium (Vector Laboratories, Burlingame CA USA). The percentage of invadopodia-forming cells was assessed by visualization on an Axioscop 2 phase-contrast/epifluorescence microscope (Carl Zeiss Inc., Toronto, ON, Canada). A total of 100 cells were counted three times per coverslip in at least three independent experiments. Cells forming ECM-degrading invadopodia were identified based on cells with at least one F-actin-enriched area of matrix degradation (characterized by loss of green fluorescence). 

### 2.10. Mutagenesis

SMAD mutant plasmid constructs incapable of SARA binding (pCMV5b-SMAD2-N381S and pCMV5b-SMAD3-N339S) [42] were generated by PCR amplification using the primers listed in Table 1. PCR-generated amplicons were purified with QIAquick kit (Qiagen), digested with XmaI and SalI-HF restriction enzymes (New England Biolabs, Whitby, ON, Canada) then ligated into the empty pCMV5b backbone using Instant sticky-end ligase (NEB). Positive clones were validated by Sanger sequencing at Centre d’Innovation Génome Québec (M.C.Gill University, Montréal, QC, Canada).

### 2.11. RNA Extraction, Reverse Transcription and Real-time qPCR

Total RNA from 1 × 10^6^ cells was extracted in TRIzol reagent (Invitrogen) according to the manufacturer’s instructions. Complementary DNA (cDNA) was synthesized using the QuantiTect Reverse Transcription Kit (Qiagen, Mississauga, ON, Canada). Quantitative real-time PCR was performed using BiMake SYBR Green master mix (BiMake) on a Rotor-Gene 3000 real-time PCR machine (Corbett Research, Kirkland, QC, Canada). Cycling conditions consisted of a 95 °C initial denaturation for 15 min, followed by 45 cycles of 15 s denaturation at 95 °C, 45 s annealing at 58 °C and 30 s elongation at 72 °C. A final elongation step of 6 min at 72 °C concluded the PCR run. The primer pair sequences used are presented in Table 2. 

### 2.12. Western Blotting and Co-Immunoprecipitation

Cells (1 × 10^6^) were serum starved for 4 h before incubation for various times under normoxic or hypoxic conditions, as described in figure legends. Protein concentration was assessed using the BCA protein assay (ThermoFisher Scientific, Ottawa, ON, Canada) according to the manufacturer’s instructions. Cells were then lysed in RIPA buffer and immunoblotting was performed as previously described [43]. For co-immunoprecipitation experiments, 5 × 10^6^ cells were lysed in NP-40 buffer supplemented with protease and phosphatase inhibitors (Roche, Mississauga, ON). Co-immunoprecipitation was performed with 500 µg of proteins incubated with primary antibody (1/100) overnight at 4 °C before the addition of protein A-agarose (Roche Applied Science, Mannheim, Germany) for 1 h at 4 °C. Immunocomplexes were then washed thrice in lysis buffer before separation on SDS-PAGE and immunoblotting. Membranes were probed overnight with primary antibodies. Anti-rabbit or anti-mouse HRP-conjugated secondary antibody or rabbit TrueBlot were used, depending on the source of the primary antibody. Peroxidase signal was revealed using the LuminataTM western HRP Chemiluminescence substrate (Millipore, Etobicoke, ON, Canada). HRP signal was captured on autoradiography film (Diamed, Missisauga, ON, Canada) and revealed using a Kodak X-Omat 200A film developer. Band intensity densitometric analysis was performed using ImageLab software (Version 6.0.0, BioRad). Original Western Blot figures shown in File S1.

### 2.13. RT^2^ Profiler PCR Array

Total RNA of 1 × 10^6^ HT-1080 cells was extracted with RNeasy-Plus mini kit (Qiagen), as directed by the manufacturer’s instructions. Reverse transcription was performed using RT^2^ First Strand kit (Qiagen) according to the manufacturer’s instructions. Using a Human Cell Motility RT^2^ Profiler PCR array (cat no. PAHS-128Z, Qiagen), 84 different genes were simultaneously amplified in 96-well plates using 500 ng of cDNA per sample, as described in the manufacturer’s instructions. Amplification and melt curves were performed on BioRad CFX96 Real-time PCR machine (BioRad), as described in the RT^2^ Profiler PCR array instructions manual. Results were analyzed using the provided RT^2^ PCR array data analysis software version 3.5 (SABiosciences). Complete normalized array results are presented in Table S1.

### 2.14. TCGA Data Analysis

Gene expression from publicly available TCGA Illumina HiSeq RNA-Seq (RSEM normalized) datasets was obtained through the TCGA Data Portal (http://cancergenome.nih.gov, accessed on 7 January 2020) [44]. The sarcoma cohort of 265 patients (SARC-TCGA, provisional) was filtered for fibroblastic sarcomas (*n* = 86; undifferentiated pleomorphic sarcomas, synovial sarcomas, myxosarcomas and desmoid/aggressive fibromatosis). The lung adenocarcinoma cohort (LUAD) was composed of 510 patients and the breast invasive carcinoma TCGA cohort of 1082 samples. Paired Spearman’s rank correlation coefficients (r) were calculated between SMAD3 target genes (*VIM, ITGB2* and *RHOB*) and tissue specific hypoxia-induced genes (*ENO1, ADM, ACOT7, CDKN3* and *TUBB6*-breast [45]; *BACH1, CAV1, LOXL2, PLOD2* and *TGFB2*-lung [46]; *ENO2, ANKRD37, GBE1, PFKB4* and *PRSS53*-fibroblastic sarcoma [47]) or common hypoxia-induced genes (*NDRG1*, *P4HA1*, *PDK1* and *SLC2A1* [45]) for each cancer patient cohort. Affymetrix gene expression results and associated overall survival and metastasis/recurrence-free survival data from sarcoma (GSE21050, [48]), lung adenocarcinoma (GSE8894, [49]) and breast [50] cancer patient cohorts were used to evaluate the correlation between *RHOB*, *VIM* and *ITGB2* gene expression and the software-optimized high- and low-risk patient groups using the publicly available online SurvExpress software (http://bioinformatica.mty.itesm.mx:8080/Biomatec/SurvivaXvalidator.jsp, accessed on 8 July 2020) [51]. 

### 2.15. Statistical Analysis

Statistical analysis was performed using GraphPad Prism software V8.0 (GraphPad, San Diego, CA, USA). Data are presented as mean ± standard error of the mean (SEM). Unpaired non-parametric Student’s *t*-test or Kruskal-Wallis ANOVA were used to assess statistical significance, unless otherwise specified. *p*-value < 0.05 was considered statistically significant.

## 3. Results

### 3.1. Hypoxia Selectively Increases pSMAD3 Levels in Different Types of Cancer 

**Figure 1:**
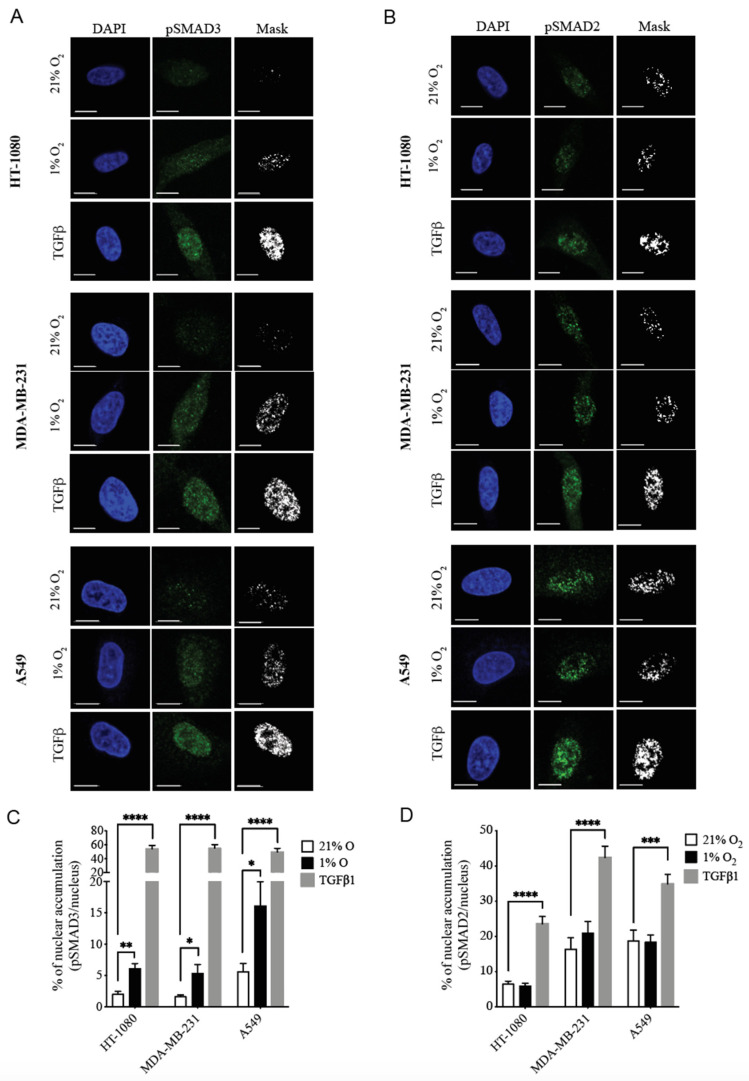
Hypoxia Selectively Increases Nuclear Accumulation of pSMAD3 in Different Cancer Cell Lines. (**A**,**B**) Representative confocal microscopy images of HT-1080, MDA-MB-231 and A549 cells incubated under normoxic (21% O_2_), hypoxic (1% O_2_) or TGFβ-supplemented conditions, stained with DAPI (blue) and anti-pSMAD3 (Ser423/425) (**A**) or anti-pSMAD2 (Ser465/467) (**B**) antibody (green). Scale bar = 10 µm. (**C**,**D**) Graphs showing the percentage of nuclear accumulation of pSMAD3 (**C**) or pSMAD2 (**D**) in HT-1080, MDA-MB-231 and A549 cells cultured under normoxic, hypoxic or TGFβ1-supplemented (1 ng/mL) conditions. (*n* = 3–5, 10 cells per group per experiment). Data are presented as mean ± SEM. * *p* < 0.05; ** *p* < 0.01; *** *p* < 0.001 and **** *p* < 0.0001.

We have previously shown that hypoxia increases the cellular level and the nuclear accumulation of phospho-SMAD3 (pSMAD3) without affecting total SMAD3 levels in HT-1080 human fibrosarcoma cells, a mechanism involved in hypoxia-induced invadopodia production. In contrast, pSMAD2 levels were not affected by the hypoxic condition [37]. To determine whether this mechanism is widespread across different types of cancer, we first evaluated the nuclear accumulation of pSMAD2 and pSMAD3 in fibrosarcoma (HT-1080), breast adenocarcinoma (MDA-MB-231) and lung carcinoma (A549) cells incubated under normoxic (21% O_2_), hypoxic (1% O_2_) or TGFβ1-supplemented conditions. Hypoxia induced a significant 2 to 3-fold increase in nuclear accumulation of pSMAD3 (Figure 1A,C) without affecting that of pSMAD2 (Figure 1B,D) in each of the cancer cell lines tested. This effect was not related to the inability of SMAD2 to be activated in these cell lines, because significant increases in pSMAD2 following stimulation with TGFβ were observed (Figure 1B,D). 

**Figure 2:**
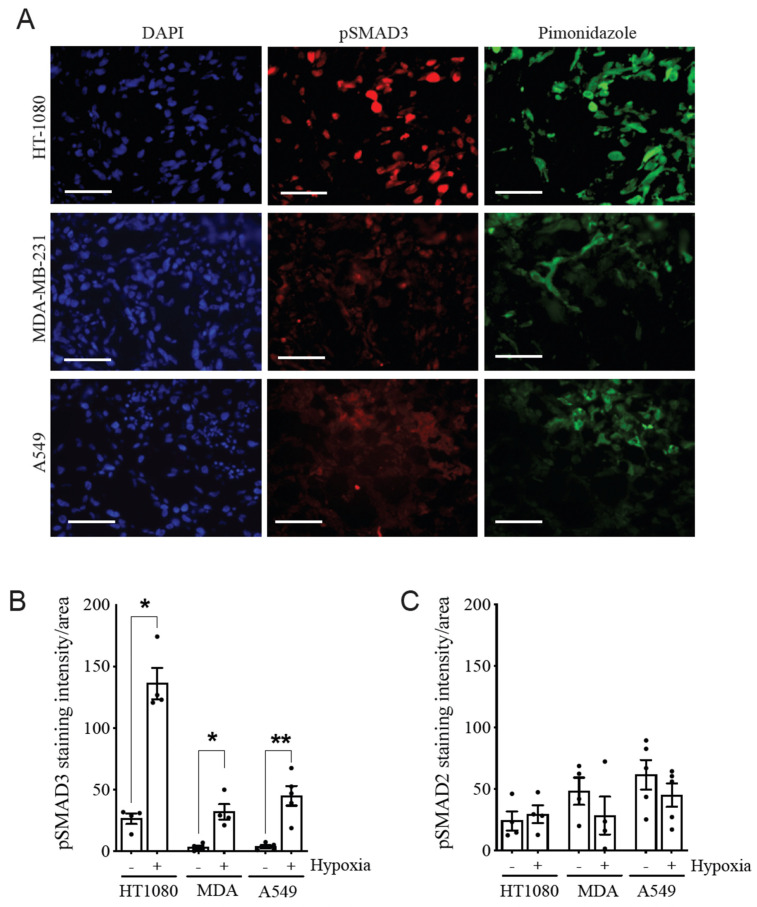
Hypoxia Selectively Increases pSMAD3 Levels in Tumor Xenografts. (**A**) Representative images of cryosections of HT-1080, MDA-MB-231 and A549 xenograft tumors, grown on CAM, stained for hypoxic areas (hypoxyprobe; red) and pSMAD3 (green). Nuclei were stained with DAPI (blue). Scale bar = 50 µm. (**B**,**C**) Quantitation of pSMAD3 (**B**) and pSMAD2 (**C**) staining intensity in normoxic (hypoxia −) and hypoxic (hypoxia +) regions. (*n* = 3–4 experiments; >4 regions analyzed in each experiment). Data are presented as mean ± SEM. * *p* < 0.05; ** *p* < 0.01.

To next evaluate the expression level of pSMAD2 or pSMAD3 in hypoxic (pimonidazole-positive) regions of tumors, the different cancer cell lines were grafted onto the chorioallantoic membrane (CAM) of chick embryos to allow them to form solid tumors. We observed significantly higher levels of pSMAD3 in pimonidazole-positive areas compared with negative areas in HT-1080-, MDA-MB-231-, and A549-derived xenografts (Figure 2A,B), whereas pSMAD2 levels were not significantly modulated (Figure 2C and Figure S1). These results demonstrate that selective activation of SMAD3 under hypoxic conditions is a common mechanism in a variety of cancers.

### 3.2. SMAD2 and SMAD3 Display Opposing Roles in Cancer Cell Invasion

**Figure 3:**
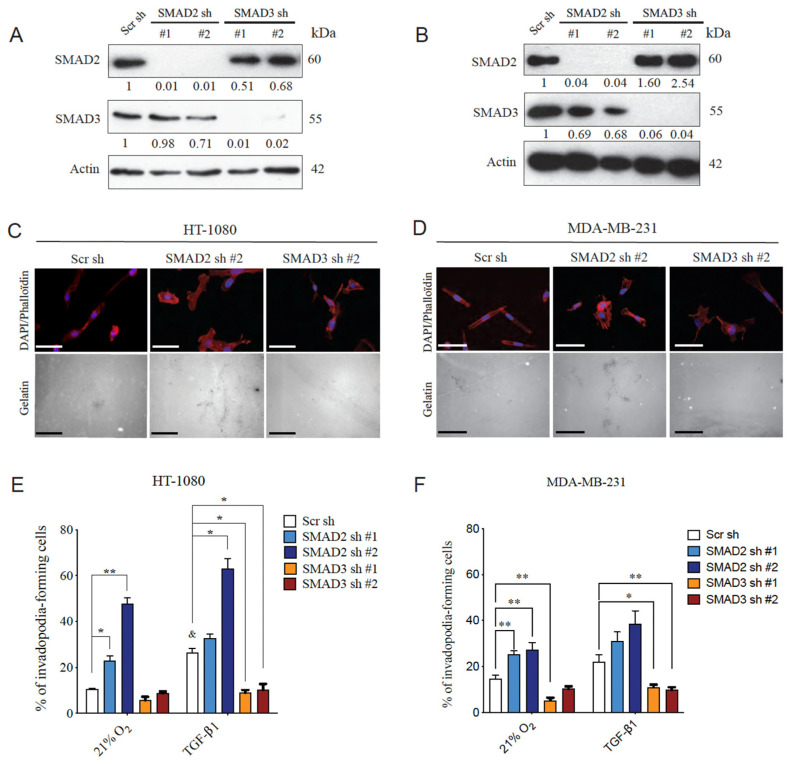
Effect of Silencing the Expression of SMAD2 or SMAD3 on Invasive Properties of Cancer Cells. HT-1080 and MDA-MB-231 cells were transduced with lentiviral shRNA against SMAD2, SMAD3 or a scrambled (Scr) shRNA. (**A**,**B**) Representative Western blot images of protein expression of SMAD2 and SMAD3 following depletion of SMAD2 or SMAD3 in HT-1080 (**A**) or MDA-MB-231 (**B**) cells. Actin was used as loading control. *n* = 3. (**C**,**D**) Representative immunofluorescence images of nucleus (DAPI:blue), actin filaments (phalloidin:red) and gelatin (grayscale) of invadopodia formation by HT-1080 (**E**) and MDA-MB-231 (**F**) cells under normoxic conditions. (**E**,**F**) Percentage of invadopodia-forming HT-1080 (**E**) and MDA-MB-231 (**F**) cells incubated for 10 h under normoxic (21%O_2_) or TGFβ1-supplemented (1 ng/mL) conditions (*n* = 3–5). Data are presented as mean ± SEM. * *p* < 0.05 and ** *p* < 0.01.

The transcription factors SMAD2 and SMAD3 have long been considered to be signaling partners with common or even redundant roles [52,53]. Although a growing body of evidence indicates that each SMAD can play distinct roles both in vitro and in vivo [23,54,55], very few studies have concomitantly compared the individual roles of SMAD2 and SMAD3 in cancer progression. One important step in tumor progression is the formation of invadopodia, a process that couples focal matrix degradation and cell movement for invasion and metastasis [56,57,58]. To compare the impact of the modulation of SMAD2 or SMAD3 expression on the invasive properties of cancer cells, we used HT-1080 and MDA-MB-231 cells overexpressing shRNA directed against SMAD2 or SMAD3. The efficacy and specificity of the shRNAs were validated by immunoblotting (Figure 3A,B). Knockdown cells were then subjected to invadopodia formation assays under normoxic condition with and without the addition of TGFβ ligand used as control. The results indicate a marked dichotomy between the effect of SMAD2 and SMAD3 inhibition on the percentage of cells producing invadopodia in both cell lines, unstimulated and stimulated with TGFβ. SMAD2 depletion markedly increased the formation of invadopodia that degrades the extracellular matrix (Figure 3C–F). Conversely, depletion of SMAD3 tends to decrease baseline invadopodia levels, an effect that was statistically significant under TGFβ stimulation (Figure 3C–F). Taken together, these results demonstrate a functional dichotomy between SMAD2 and SMAD3, where inhibition of SMAD2 promotes the production of cell invasion structures, whereas SMAD3 depletion prevents it.

### 3.3. SMAD3 Is Essential for Hypoxia-Induced Cell Invasion and Tumor Progression

**Figure 4:**
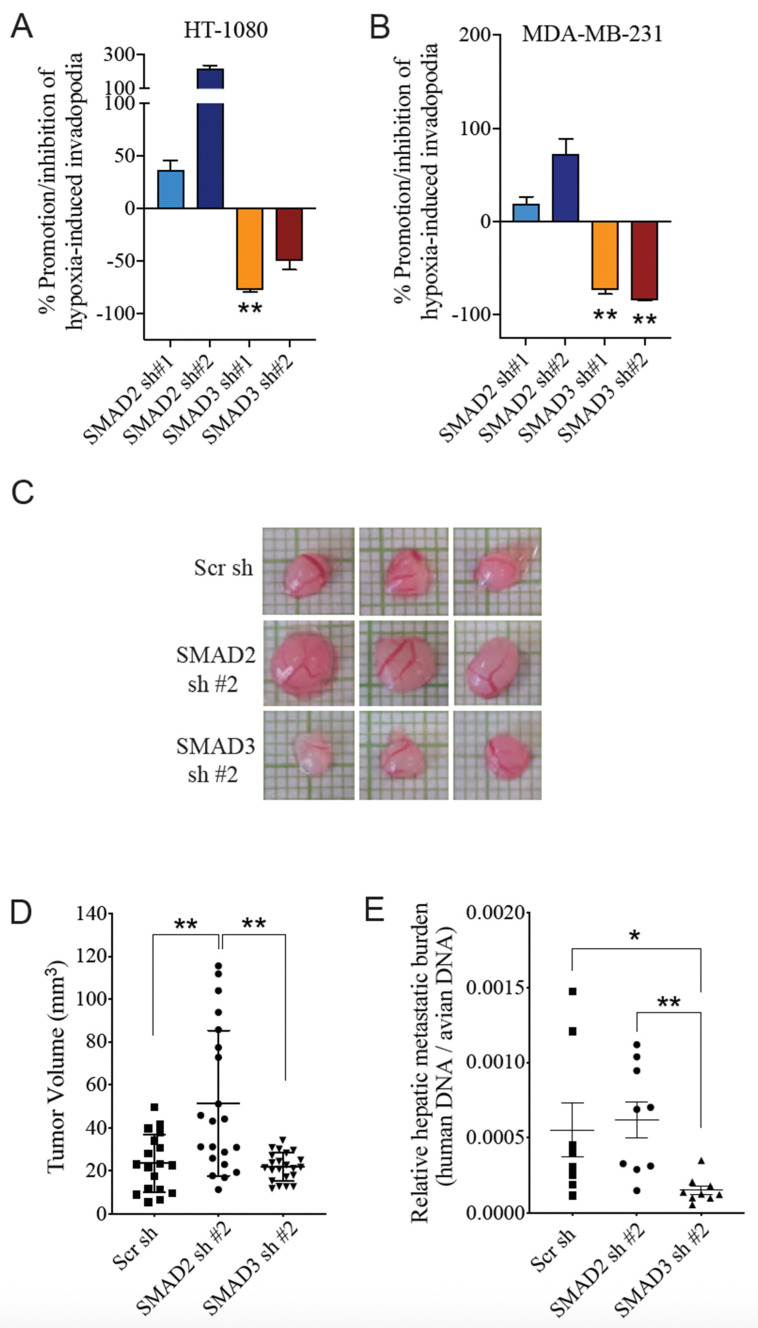
Effect of SMAD2 and SMAD3 Depletion on Hypoxia-Induced Cancer Cell Invasion. (**A**,**B**) Percentage of promotion/inhibition of hypoxia-induced invadopodia formation by HT-1080 (**A**) and MDA-MB-231 (**B**) cells, compared to Scr sh. (**C**–**E**) HT-1080 cells transduced with scrambled, SMAD2- or SMAD3- targeted shRNA were grown on chicken chorioallantoïc membrane (CAM) for 7 days. (**C**) Representative images of xenograft tumors obtained. Xenograft tumor size (**D**) and relative amount of spontaneous liver metastasis in the chicken embryo (**E**) were quantitated (*n* = 20–24 per group). Data are presented as mean ± SEM. * *p* < 0.05; ** *p* < 0.01.

To next evaluate the impact of SMAD2 or SMAD3 modulation on the invasive properties of cancer cells in hypoxia, we compared the capacity of HT-1080 and MDA-MB-231 knockdown cells to produce invadopodia under normoxic versus hypoxic conditions. Depletion of SMAD3 significantly reduced the ability of cells to produce invadopodia in response to hypoxia, whereas this ability was retained in SMAD2-depleted cells (Figure 4A,B). This is consistent with the observation that SMAD3 remains intact (thereby available for hypoxic response) in SMAD2-depleted cells (Figure 3A,B). These results suggest a selective role for SMAD3 in hypoxia-induced cell invasion. We have previously shown that the CAM xenograft assay recapitulates the pro-invasive hypoxic microenvironment of solid tumors [59]. Using this xenograft model, we observed a difference in the size of tumors derived from HT-1080 cells transduced with shRNAs directed against SMAD2 or SMAD3 (Figure 4C). SMAD2-depleted xenografts had a significantly larger volume than control tumors, and their volume was, on average, twice that of tumors in which SMAD3 expression was suppressed (Figure 4D). Furthermore, and in agreement with the results of invadopodia formation assays, the relative number of spontaneously metastasized cells to chicken embryo livers was significantly higher in cells depleted of SMAD2 compared to the reduction observed in SMAD3-depleted cells (Figure 4E). Overall, these findings further emphasize the SMAD2/3 dichotomy in tumor progression and suggest that SMAD3 expression is important for cancer cell invasion in response to hypoxia.

### 3.4. SMAD3-SARA Binding Is Important for Hypoxia-Induced Invadopodia Production

**Figure 5:**
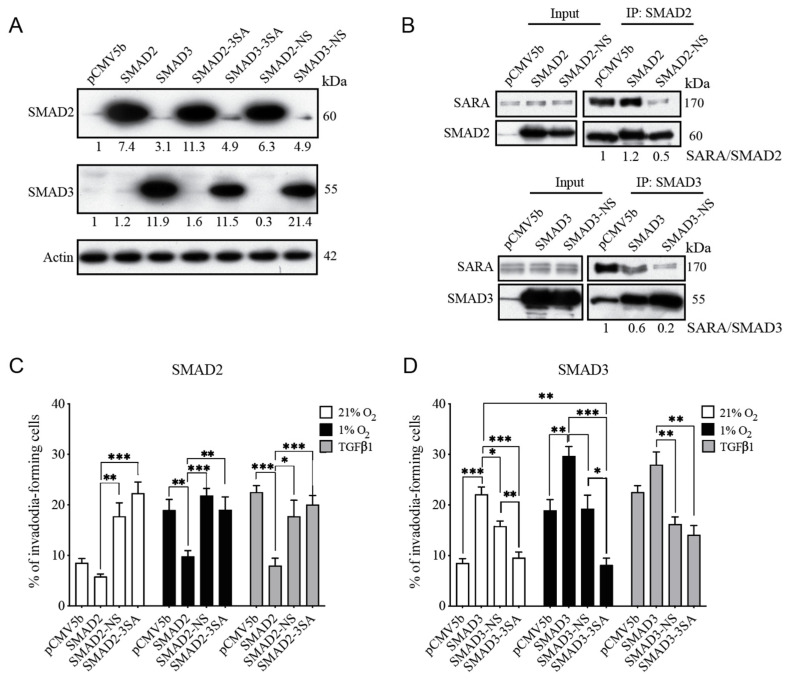
SMAD2 and SMAD3 Depend on SARA Binding to Modulate Cancer Cell Invasion. HT-1080 cells were transiently transfected with wild-type, C-terminal mutant (3SA), or SARA-binding mutant N339/381S (NS) SMAD2 or SMAD3 constructs, using empty vector (pCMV5b) as a control. (**A**) Representative Western blot showing overexpression specificity and efficacy of each construct. (**B**) Representative co-immunoprecipitation assays showing the reduction of SARA-binding to each SMAD NS constructs. Densitometric analysis of co-IP SARA/SMAD2 or SMAD3 IP was performed. **C**,**D**) Percentage of invadopodia-forming HT-1080 cells transfected with SMAD2 (**C**) or SMAD3 (**D**) constructs after 10 h incubation under normoxic (21% O_2_), hypoxic (1% O_2_) or TGF-β1 supplemented (1 ng/mL) condition (*n* = 3–5). Data are presented as mean ± SEM. * *p* < 0.05; ** *p* < 0.01; *** *p* < 0.001.

The adaptor protein SMAD Anchor for Receptor Activation (SARA) has been shown to facilitate cytosolic SMAD2/3 recruitment to TGFBR1 [60], which allows SMAD2/3 C-terminal phosphorylation, a necessary step for their nuclear accumulation and subsequent role in the regulation of gene expression. The importance of SARA in SMAD2 C-terminal phosphorylation has been well documented [60,61,62], but its implication in SMAD3 activation remains controversial [63,64]. To determine whether the SMAD2/3 dichotomy found herein depends on a SARA-driven activation cascade, HT-1080 cells were transfected with wild-type or N339/381S (NS) mutant SMAD2/3 constructs, the latter share a point mutation that prevents their binding to SARA. Accordingly, these mutants fail to synergize with SARA to potentiate TGFβ signaling but can still bind and be activated by TGFBR1 independently of SARA [42]. The C-terminal SMAD2/3 mutant (3SA) that cannot be activated by TGBR1 was used as a negative control of SMAD activation [38]. Validation of the overexpression efficiency of each construct was performed by immunoblotting (Figure 5A) and the inhibition of the interaction of SARA with SMAD2-NS and SMAD3-NS constructs was confirmed by co-immunoprecipitation assays (Figure 5B). Cells overexpressing these constructs were then submitted to invadopodia formation assays under normoxic or hypoxic conditions. TGFβ1 stimulation was used as a positive control of SMAD activation. Further supporting its role as a suppressor of cell invasion, overexpression of SMAD2 tends to decrease the percentage of cells producing invadopodia, an effect that was more prominent in cells cultured under hypoxic or TGFβ1-supplemented conditions (Figure 5C). In contrast, the SARA binding-deficient SMAD2-NS mutant failed to suppress invadopodia formation in cells cultured under normoxic, hypoxic, or TGFβ1-supplemented conditions (Figure 5C). Consistent with the opposing roles of SMAD2/3 in cell invasion, overexpression of SMAD3 promoted invadopodia formation in all culture conditions (Figure 5D). This effect was lost in cells expressing the SMAD3-NS mutant (Figure 5D). In addition, SMAD3-3SA had a more pronounced effect in reducing invadopodia production than the NS construct in normoxic and hypoxic conditions, consistent with the inability of the 3SA construct to be activated by the TGFBR1. Collectively, these results suggest that SMAD2 and SMAD3 depend on an interaction with SARA to mediate their anti- and pro-invasive functions, an effect found in different culture conditions, including hypoxia.

### 3.5. Hypoxia Selectively Increases the Interaction between SARA and SMAD3, an Event Linked to Increased SMAD3 Bioavailability

**Figure 6:**
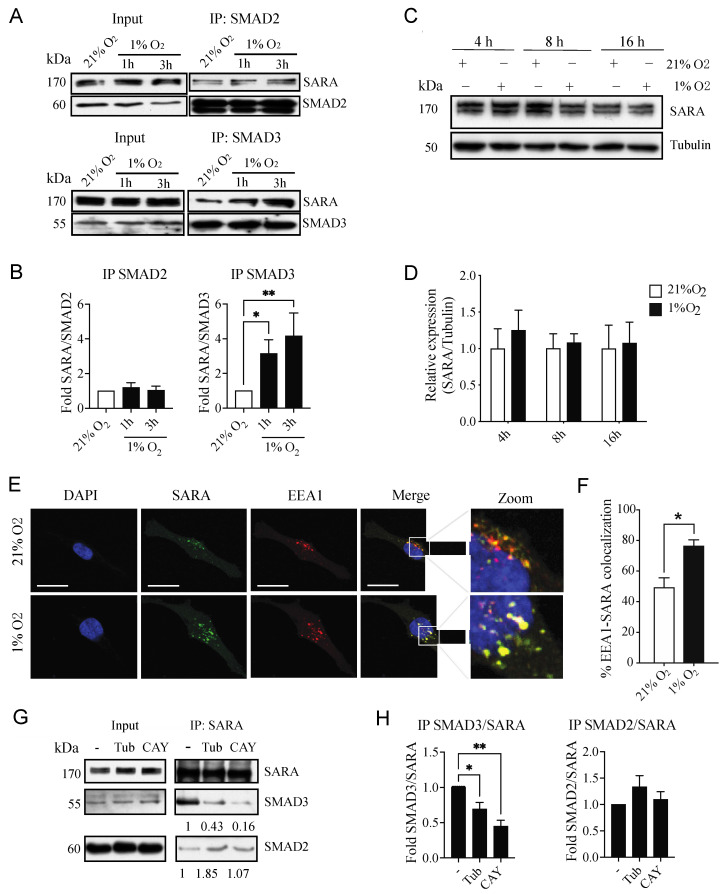
Effect of Hypoxia and HDAC6 Activity on SMAD3/SARA Interaction. (**A**,**B**) Co-immunoprecipitation of SMAD2 or SMAD3 from HT-1080 cells incubated under normoxic (21% O_2_) or hypoxic (1% O_2_) condition. Representative Western blot images (**A**) and densitometric analysis (**B**) are presented (*n* = 4–5). (**C**,**D**) HT-1080 cell-derived proteins were extracted after increasing incubation period under hypoxic conditions, then immunoblotted for SARA and tubulin, as loading control. Representative western Blot images (**C**) and densitometric quantitation of relative SARA protein expression (**D**) are presented (*n* = 3). Data are presented as mean ± SEM. * *p* > 0.05; ** *p* < 0.01. (**E**,**F**) Immunofluorescence assay of HT-1080 cells stained for DAPI (nucleus; blue), SARA (green) and EEA1 (red). Representative confocal images (**E**) and quantitation of SARA/EEA1 colocalization (**F**) are presented (*n* = 3, >10 cells per experiment). Data are presented as mean ± SEM. * *p* > 0.05. (**G**,**H**) HT-1080 cells were treated with the HDAC6 inhibitors tubacin or CAY10603, then incubated for 3 h under hypoxic conditions. (**G**) Representative co-immunoprecipitation of SARA, immunoblotted for SARA, SMAD3 and SMAD2, in hypoxic conditions (1% O_2_) in the presence or absence of Tubacin (Tub; 10 µM) or CAY10603 (CAY; 1 µM). (**H**) Densitometric analysis of co-IP SMAD2 or SMAD3/SARA IP was performed (*n* = 6). Data are presented as mean ± SEM. * *p* > 0.05; ** *p* < 0.01.

Because both SMAD2 and SMAD3 require interaction with SARA to modulate invadopodia formation, we next sought to determine why only SMAD3 was essential for hypoxia-induced invadopodia formation by examining the interaction between each SMAD and SARA. To do so, we performed co-immunoprecipitations of SARA, SMAD2 or SMAD3 from cell lysates of HT-1080 cells incubated under normoxic or hypoxic conditions. The results indicate that the interaction between SMAD2 and SARA was not significantly modulated by hypoxia, whereas the interaction between SMAD3 and SARA was increased in a time-dependent manner (Figure 6A,B and Figure S2A).

SMAD signaling through SARA/SMAD interaction depends on three main aspects: SARA abundance, SARA localization, and SMAD abundance/availability. To evaluate the effects of hypoxia on SARA expression levels, we performed immunoblotting and qPCR quantitation of SARA expression in cells exposed to normoxic or hypoxic conditions for different time-periods. The results indicated no significant modulation of SARA protein (Figure 6C,D) or mRNA (Figure S2B,C) expression in hypoxic cancer cell lines.

Because hypoxia is known to alter the subcellular localization of various proteins [33,39,65,66], we next measured the percentage of colocalization between SARA and Early Endosome Antigen 1 (EEA1), a marker of early endosomes, which corresponds to the cellular compartment where SARA is known to facilitate SMAD2- and SMAD3-dependent TGFβ signaling [67,68,69]. Despite a strong colocalization of SARA and EEA1 in normoxia (49%), hypoxia further increased the recruitment of SARA to EEA1-labeled endosomes by 27% (Figure 6E,F).

SMAD availability can be affected by protein expression levels as well as cytoskeleton sequestration [26,70]. We have previously demonstrated that hypoxia did not affect total SMAD2 or SMAD3 protein levels but augmented SMAD3 bioavailability through the activation of a cytosolic histone deacetylase 6 (HDAC6)-dependent tubulin deacetylation pathway [37]. HDAC6 activity was also reported to be important for increasing SMAD3 phosphorylation in response to TGFβ [71]. We therefore reasoned that the increased recruitment of SMAD3 to SARA under hypoxic conditions might be due, at least in part, to HDAC6-induced increased SMAD3 bioavailability. This event would give SMAD3 an advantage for SARA binding, as SMAD2 and SMAD3 share a conserved SARA binding region [42]. Treatment of cells with selective HDAC6 inhibitors, tubacin and CAY10603, whose activity was demonstrated by their ability to prevent tubulin deacetylation (Figure S3), significantly decreased the binding of SMAD3, but not SMAD2, to SARA under hypoxic conditions (Figure 6G,H). So far, these data suggest that hypoxia selectively increases the SMAD3 activation pathway through increased SARA recruitment to EEA1+ endosomes and increased HDAC6-dependent SMAD3 bioavailability, in addition to SARA/SMAD3 binding.

### 3.6. Pharmacological Inhibition of HDAC6 and SMAD3 Impedes Tumor Progression

**Figure 7:**
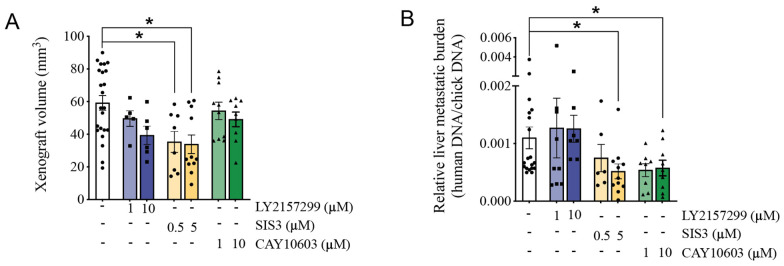
Effect of HDAC6 Inhibition on Tumor Progression. (**A**,**B**) HT-1080 cells treated with inhibitors selective for TGFBR (LY215799), SMAD3 (SIS3), or HDAC6 (CAY10603) or control vehicle were grown on CAM for 7 days. Tumor xenograft volume (**A**) and chick embryo liver metastatic burden (**B**), as measured by primate Alu-sequence repeats expression relative to chick GAPDH expression, were quantitated. Data are presented as mean ± SEM. Each dot represents one tumor xenograft. * *p* < 0.05.

To next investigate the relative contribution of the HDAC6-SARA-SMAD3 axis in tumor progression, HT-1080-derived tumors were treated with pathway-specific inhibitors in the CAM xenograft assay. Only xenografts treated with the SMAD3 inhibitor SIS3 showed a significant reduction in volume compared with xenografts treated with vehicle (Figure 7A). In addition, significant inhibition of metastatic load to the chick embryo liver was observed when xenografts were treated with SIS3 or the HDAC6 inhibitor CAY10603 but not with the TGFBR inhibitor, LY2157299 (Figure 7B), which is consistent with the mixed results observed with this compound in clinical trials [12,72,73]. These results suggest an advantage for the selective inhibition of the pro-invasive HDAC6-SMAD3 axis over global inhibition of the TGFBR signaling pathway in order to limit tumor progression.

### 3.7. Selective SMAD3-Modulated Genes Participate in Hypoxia-Induced Invadopodia Formation

**Figure 8:**
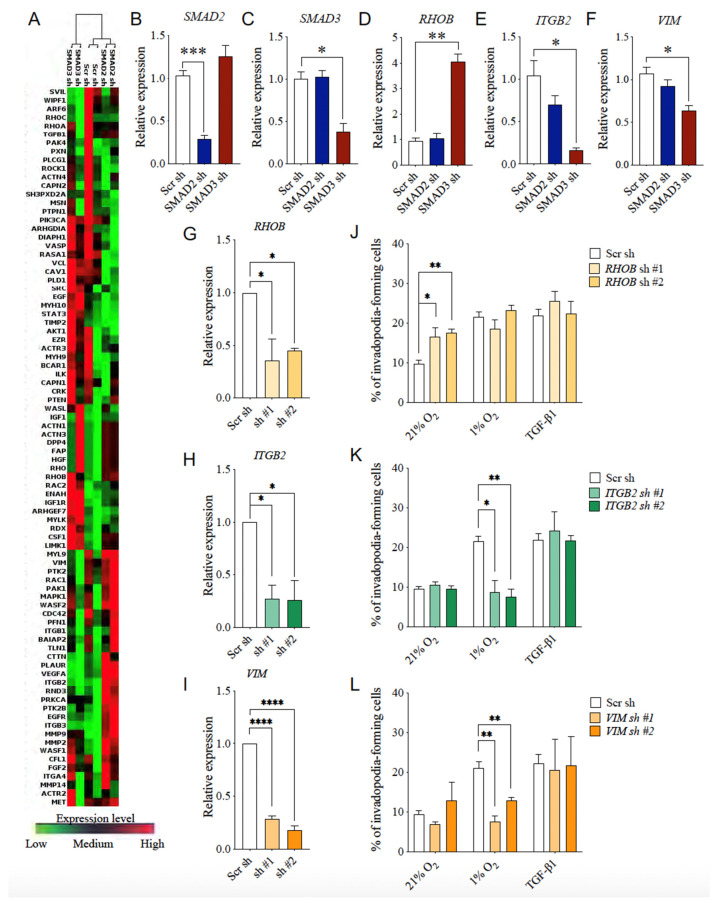
SMAD3 Target Genes Implication in Invadopodia Production by Cancer Cells. (**A**) Clustergram generated by RT^2^ PCR array data analysis software showing expression level of 84 cell invasion-related genes. (**B**–**F**) Bar graph showing relative expression of *SMAD2* (**B**), *SMAD3* (**C**) and SMAD3 target genes *RHOB* (**D**), *ITGB2* (**E**), *VIM* (**F**) in HT-1080 cells transduced with SMAD2- or SMAD3-targeted shRNAs (*n* = 3). (**G**–**L**) HT-1080 cells were transduced with shRNAs targeting the individual SMAD3 invasion-related genes *RHOB*, *ITGB2* and *VIM*, and submitted to invadopodia assays under normoxic (21% O_2_), hypoxic (1% O_2_), or TGFβ1-supplemented conditions. Relative expression (**G**–**I**) and percentage of invadopodia-forming cells (**J**–**L**) following each gene depletion are presented (*n* = 3–6). Data are presented as mean ± SEM. * *p* < 0.05; ** *p* < 0.01; *** *p* < 0.001; **** *p* < 0.0001.

To better understand the selective SMAD3-mediated promotion of cell invasion, we investigated the effect of inhibiting the expression of SMAD2 or SMAD3 on the level of expression of genes involved in cell motility, using a PCR array approach. The expression level of 84 invasion-related genes in cells lacking SMAD2 or SMAD3 compared with control cells (Scr sh) are shown in Figure 8A. Unsupervised clustering of the data dissociated the gene expression profile of SMAD3-depleted cells from that of control cells and SMAD2-depleted cells (Figure 8A). Differential expression analysis identified five genes whose expression was significantly positively or negatively modulated by more than 1.5-fold in response to SMAD3 depletion, compared to control cells (Table 3). Among these genes, *RHOB*, *ITGB2* and *VIM* were selectively modulated by SMAD3 depletion, while *CSF1* and *ARF6* were affected by the depletion of SMAD2 or SMAD3. The selective modulation of *SMAD2* and *SMAD3,* and that of the SMAD3-selective target genes, namely *RHOB*, *ITGB2* and *VIM*, were validated by qPCR in HT-1080 cells transduced with shRNA directed against *SMAD2* or *SMAD3* (Figure 8B–F).

To determine the contribution of individual SMAD3 target genes to cell invasion in hypoxia, HT-1080 cells were transduced with *RHOB*, *ITGB2* or *VIM*-targeted shRNAs and submitted to invadopodia-formation assays under various culture conditions. Validation of shRNA efficiency for each target was performed by qPCR (Figure 8G–I). A significant increase in cancer cell invasion under normoxic conditions was observed in *RHOB*-depleted cells, compared to control cells. In contrast, *RHOB* depletion did not affect invadopodia formation in cells stimulated with TGFβ1 or incubated under hypoxic conditions (Figure 8J). Interestingly, *ITGB2* or *VIM* depletion selectively abrogated hypoxia-induced invadopodia production in HT-1080 cells (Figure 8K–L), suggesting that their expression is necessary for hypoxia-induced invadopodia production. In addition, the use of ITGB2 blocking antibodies induced a dose-dependent reduction in hypoxia-induced invadopodia production by HT-1080 and MDA-MB-231 cells, whereas no significant modulation was observed under TGFβ-supplemented conditions (Figure S4), suggesting that both *ITGB2* expression and its integrin activity are involved in hypoxia-induced invadopodia production.

**Figure 9:**
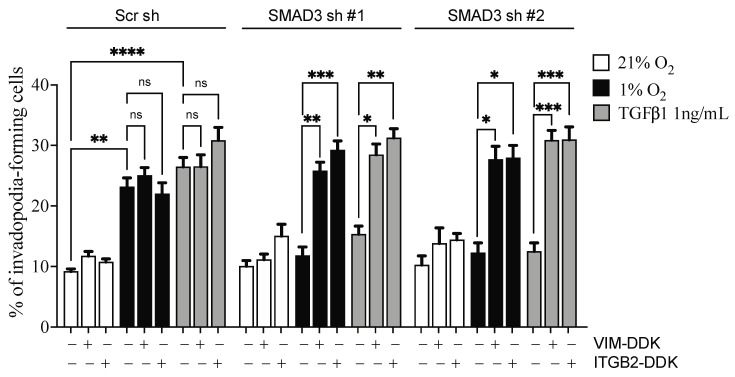
Overexpression of VIM or ITGB2 Rescues Invadopodia Production in SMAD3-Depleted Cells. HT-1080 cells were stably transduced with control shRNA (Scr sh) or shRNAs targeting SMAD3, and transfected with *ITGB2* or *VIM* overexpression constructs, using empty vector construct as a control. Cells were then submitted to invadopodia assays under normoxic (21% O_2_), hypoxic (1% O_2_), or TGFβ1-supplemented conditions. Percentage of invadopodia-forming cells are presented (*n* = 3–4 independent experiments). Data are presented as mean ± SEM. * *p* < 0.05; ** *p* < 0.01; *** *p* < 0.001, and **** *p* < 0.0001.

To establish the importance of VIM and ITGB2 in the SMAD3 pro-invasive pathway exacerbated by hypoxia, we performed rescue experiments in SMAD3-depleted cells which were submitted to invadopodia assays. SMAD3 depletion and VIM or ITGB2 overexpression were confirmed by Western blotting (Figure S5). The results demonstrated that hypoxia-induced invadopodia production in SMAD3-depleted cells was recovered by overexpression of either VIM or ITGB2 (Figure 9). Similar results were obtained in the TGFβ1-supplemented condition. Altogether, these results support a role for *ITGB2* and *VIM* in the SMAD3-dependent pro-invasive properties of cancer cells that are accentuated by hypoxic conditions.

### 3.8. SMAD3 Proinvasive Gene Signature Correlates with a Hypoxic Gene Signature and Prognosis in Cancer Patients

**Figure 10:**
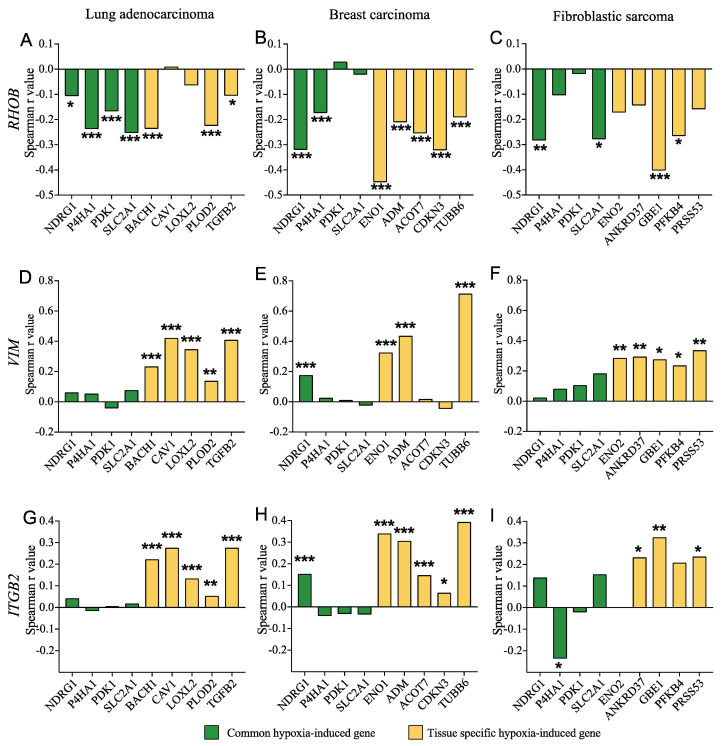
SMAD3-Selective Target Genes Correlate with Hypoxia-Induced Genes in Cancer Patients. TCGA RNA-seq data sets from sarcoma, lung adenocarcinoma and invasive breast cancer patient cohorts were used to assess the correlation coefficient between *RHOB* (**A**–**C**), *VIM* (**D**–**F**) or *ITGB2* **(G**–**I)** and genes known to be induced by hypoxia in lung adenocarcinoma (**A**,**D**,**G**), breast carcinoma (**B**,**E**,**H**) and fibroblastic sarcoma (**C**,**F**,**I**) patient cohorts. * *p* < 0.05; ** *p* < 0.01; *** *p* < 0.001.

To study the concordance between the proinvasive SMAD3 gene signature and intratumoral hypoxia, we used publicly available RNA sequencing data and assessed the correlation between the expression of *RHOB*, *VIM* and *ITGB2* and that of known hypoxia-induced genes in lung adenocarcinoma [46], breast carcinoma [45] and fibroblastic sarcoma [47]. Using the Spearman correlation index, we observed, as expected, that *RHOB* expression negatively correlated with the hypoxia-induced gene signature in lung, breast and fibrosarcoma tumors (Figure 10A–C), while *VIM* and *ITGB2* expression were generally positively correlated in all three cancer patient cohorts (Figure 10D–I). Consistent with the finding that hypoxic gene signatures are more robust if they are tumor site specific [74], such a correlation was more prevalent with tissue-specific than with common hypoxia-inducible genes.

**Figure 11:**
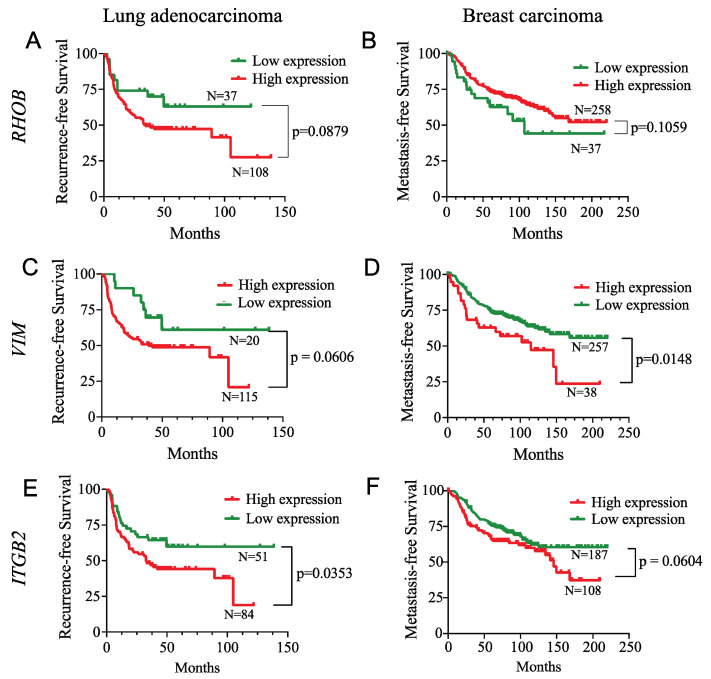
SMAD3 Invasion-Related Gene Signature Correlates with Lung and Breast Cancer Progression. Lung adenocarcinoma and breast carcinoma cancer patient cohorts were segregated into high- and low- risk groups for metastasis/recurrence-free survival using SurvExpress online software. Kaplan–Meier curves for high- (red) and low- (green) *RHOB* (**A**,**B**), *VIM* (**C**,**D**) or *ITGB2* (**E**,**F**) gene expression in lung adenocarcinoma (**A**,**C**,**E**) and breast carcinoma (**B**,**D**,**F**) human tumors are presented. Gehan–Breslow–Wilcoxon test was performed to assess *p*-value.

In addition, we asked whether the expression of the SMAD3 gene signature was correlated with tumor progression, as defined by metastasis-free survival in breast cancer and progression-free survival in lung adenocarcinoma. Using the SurvExpress web tool [51], we found that *RHOB* expression had conflicted predictive value for cancer progression (Figure 11A,B), whereas high *VIM* and *ITGB2* expression were positively correlated with low metastasis-free/recurrence-free survival in lung adenocarcinoma (Figure 11C,E) and breast cancer (Figure 11D,F) cohorts. We were unable to perform a similar correlation study with fibroblastic sarcoma patients because this cohort was not available for analysis. Overall, these results suggest that the SMAD3-dependent gene signature is present in hypoxic tumors and that *VIM* and *ITGB2* are better predictors of metastasis or cancer recurrence in lung carcinoma and breast cancer than *RHOB*.

## 4. Discussion

In this study, we have shown that SMAD2 and SMAD3 exert distinct effects on cancer cell invasion. SMAD2 expression correlated negatively and SMAD3 expression positively with the invasive capacity of cancer cells, both in vitro and in vivo. While both SMAD2 and SMAD3 signaling were detected under hypoxic conditions, only the pro-invasive SMAD3 pathway was exacerbated by the hypoxic microenvironment. Hypoxia-induced activation of the SMAD3 pathway through increased SMAD3 bioavailability and recruitment to the adapter protein SARA induced tumor progression and cancer cell invasion, events associated with the expression of SMAD3 gene targets *ITGB2* and *VIM*. Furthermore, we demonstrated that there was a significant advantage to selectively blocking the SMAD3 signaling pathway rather than inhibiting global TGFBR signaling to prevent tumor progression and metastasis.

The tumor suppressor function of SMAD2 [18,22,75,76] and the pro-tumorigenic role of SMAD3 [16,17,22,77] have already been demonstrated in various models, but only a few studies have been conducted to assess the selective importance of SMAD2 vs. SMAD3 in cancer progression. We described herein the dichotomous role of SMAD2 and SMAD3 in the ability of cancer cells to form invadopodia and to produce spontaneous metastasis. More precisely, we demonstrated that the tumor suppressive function of SMAD2 remained intact while the pro-invasive role of SMAD3 was exacerbated under hypoxic conditions. Although we showed that SMAD2 depleted cells generated larger tumor xenografts, a previous study has shown that selective knockdown of SMAD2 inhibited cell growth in vitro [22]. However, the same study also demonstrated that, despite the reduction in proliferation of SMAD2-deficient cells observed in vitro, the size of the generated metastases was significantly greater [22]. This last finding is consistent with the increase in spontaneous liver metastasis generated by SMAD2 knockdown cells compared with SMAD3-depleted cells observed in this study. Furthermore, low levels of SMAD2 activation were positively correlated with reduced overall survival in ductal carcinoma of the breast [78] and increased cancer invasion in gastric cancer [79]. Conversely, increased SMAD3 expression was associated with shorter overall survival in esophageal squamous cell carcinoma [20] and gastric cancer [80]. Taken together, these findings suggest that cancer progression is related to increased pro-tumorigenic SMAD3 signaling, which prevails over SMAD2 signaling in the hypoxic tumor microenvironment. It is likely that part of the mechanism by which hypoxia activates SMAD3 signaling involves autocrine production of TGFβ, as previous findings have indicated that hypoxia increases the expression of the TGFβ pro-protein convertase furin in cancer cells, resulting in enhanced processing and bioactivation of the TGFβ precursor form [34]. This event was then implicated in hypoxia-induced invadopodia formation [37].

The mechanistic basis of the SMAD3 bias proposed herein includes a hypoxia-induced increase in SMAD3-SARA binding that relies on HDAC6-dependent SMAD3 bioavailability, together with an increased recruitment of SARA to EEA1+ endosomal vesicles. It is known that TGFBR signaling is induced following its internalization into endosomal compartments [67,68,69]. The SARA protein contains a FYVE domain that binds to phosphatidyl-inositol-3-phosphate (PI3P), a phosphoinositide highly enriched in early endosomes where SARA-dependent SMAD signaling is known to occur [81]. Since the affinity of FYVE domains for PI3P has been described to increase in acidic pH [82], and since hypoxia is known to acidify vesicular pH [39,83], pH-dependent changes in SARA affinity for PI3P would be a potential explanation for the increased recruitment of SARA to EEA1+ endosomal vesicles found in this study.

SARA was initially described as an adaptor protein facilitating the recruitment of SMAD2/3 to TGFBR1 [60]. Since then, several discrepancies have been published regarding the importance of SARA in SMAD-dependent TGFβ signaling. One study has shown that the depletion of SARA, or deletion of its SMAD Binding Domain (SBD) in endothelial cells, inhibited the transcriptional activity of both SMAD2 and SMAD3 in vitro [84], whereas a separate study has shown that SARA depletion in HeLa cells or Mv1Lu cells had little effect on these SMADs [64,85]. SARA was also shown to inhibit SMAD2/3 signaling in rat neural cells [86]. As alternatives to the targeting of TGFβ or TGFBR per se, different research groups have developed peptide aptamers of SARA SBD that have shown significant inhibition of SMAD2/3 signaling in human renal epithelial cells and mouse mammary gland cells [87,88]. However, this strategy has failed to progress to clinical trials, possibly due to the lack of studies on the characterization of SARA-dependent SMAD signaling in tumor progression. Our results suggesting that SARA is an active participant in both SMAD2-inhibitory and SMAD3-stimulatory effects on cancer cell invasion are consistent with the possibility that the targeting of SARA/SMAD binding would be unlikely to provide significant therapeutic benefits for cancer patients.

To date, no selective biomarkers of the pro-tumorigenic axis of TGFβ signaling have been uncovered, but it is possible that further study of the SMAD3 signaling axis promoted by hypoxia could lead to the identification of candidate genes. As critical regulators of TGFβ-induced gene transcription, SMAD2 and SMAD3 can interact with distinct transcription factors to regulate selective gene subsets (reviewed in [15]). Among the 84 cell motility genes studied here, *VIM* and *ITGB2* were found to be selective targets of SMAD3, and these two genes strongly correlated with the tissue-specific hypoxic gene signature in fibrosarcoma, breast carcinoma and lung adenocarcinoma cancer types, suggesting their regulation by the exacerbated SMAD3 pathway in various tumors. Using knockdown and rescue experiments, we demonstrated that *VIM*, in addition to *ITGB2*, expression was involved in hypoxia-induced invadopodia formation. We also demonstrated that these genes are good predictors of cancer metastasis or recurrence in cohorts of breast and lung cancer patients. These results are in agreement with previous studies demonstrating that high expression of *VIM* or *ITGB2* is related to cancer progression and metastasis [89,90,91,92,93]. In addition, hypoxia [94,95,96] and TGFβ [97,98,99,100] have been shown to induce *VIM* and *ITGB2* expression. Although the effect of hypoxia on ITGB2 activity has not yet been described, hypoxia is known to be involved in the routing, recycling, and activation of various integrin β subunits [101,102,103], suggesting that it may also have an effect on ITGB2 activity. Taken together, these data suggest that *VIM* and *ITGB2* are potential biomarkers of the SMAD3-dependent pro-invasive axis exacerbated by hypoxia.

Due to the central role of TGFβ in tumor progression, numerous compounds aimed at blocking TGFβ signaling have been tested in clinical trials, but none have yet been approved for clinical use. The compounds that are now undergoing clinical testing are mainly ligand traps (AVID200, Luspatercept), blocking antibodies (Fresolisumab), antisense oligonucleotides (Trabedersen) and TGFBR kinase inhibitors (Vactosertib and Galunisertib) [10,104,105]. These treatment strategies often generate serious adverse effects due to the pleiotropic functions of TGFβ in normal and cancer cells and have mitigated effects on tumor progression, perhaps because they do not consider the dual role of TGFβ in cancer. Our results demonstrating that selective inhibition of SMAD3 or HDAC6-dependent SMAD3 bioavailability was more potent than Galunisertib (LY2157299) in inhibiting tumor growth and metastasis, combined with those indicating a significant increase in SMAD3 activation in the hypoxic tumor microenvironment, suggest that SMAD3 can be an interesting therapeutic target for impeding cancer progression. Selective SMAD3 inhibition has not been extensively explored and only a few SMAD3-targeting compounds have been developed. In this regard, the small molecule SIS3 [106], cell-permeable peptides impeding pSMAD3 nuclear localization [107], and the natural flavonoid Naringenin [108] have shown promising therapeutic effects in diabetes, pulmonary fibrosis, heart disease, lung cancer and melanoma animal models [107,109,110,111]. However, none of these compounds have reached clinical trials, stressing the need for further development of these compounds.

## 5. Conclusions

In the present study, we described how hypoxia selectively induced the pro-invasive arm of TGFβ signaling through activation of a HDAC6- SMAD3-SARA-ITGB2/VIM pathway that contributes to the ability of cancer cells to invade and form metastases. In addition, we demonstrated a significant advantage in selectively inhibiting the SMAD3 signaling pathway rather than inhibiting global TGFBR signaling to prevent tumor progression and metastasis. Altogether, our results suggest that fine-tuning of the TGFBR-SMAD3-associated axis rather than complete inhibition of TGFβ signaling would be a better strategy for cancer treatment. However, confirmation of the direct link between the hypoxic HDAC6-SMAD3-SARA pathway and the ITGB2/VIM effect on hypoxia-induced cancer cell invasion will require further investigation.

## Figures and Tables

**Table 1:** Primer sequences used for the generation of SMAD-NS mutants.

Construct	Primer Sequence
**pCMV5b-SMAD2-N381S**	
Forward	CAAGGGTCGACAATCGTCCATCTTG
Mutant forward	GTTCATCTTCAGACTACAGCCTGGTGG
Mutant reverse	CCACCAGGCTGTAGTCTGAAGATCAAC
Reverse	GATGCCACCCGGGTCTAGATTATGAC
**pCMV5b-SMAD3-N339S**	
Forward	CAAGGGTCGACTGTCGTCCATC
Mutant forward	GATCTTCAGGCTGCATCCTGGTG
Mutant reverse	CACCAGGATGCAGCCTGAAGATC
Reverse	GATGCCACCCGGGTCTACACTAAG

Underlined bases contain the desired mutation.

**Table 2:** Primer sequences used for quantitative real-time PCR.

Gene	Forward Primer Sequence	Reverse Primer Sequence
SMAD2	CGAAATGCCACGGTAGAAATG	GGGCTCTGCACAAAGATTG
SMAD3	TCCATCCCCGAAAACACTAAC	CATCTTCACTCAGGTAGCCAG
RHOB	ATCCAAGCCTACGACTACCT	AGTTGATGCAGCCGTTCT
ITGB2	GTGAACACGCACCCTGATAA	GGAGTTGTTGGTCAGCTTCA
VIM	GATTCACTCCCTCTGGTTGATAC	GTCATCGTGATGCTGAGAAGT
RPLP0	GATTACACCTTCCCACTGC	CCAAATCCCATATCCTCGTCCG
SARA	GGATTCTCAGGCTCCAAATTGC	GGCTGAGCCACATTCATTAGC

**Table 3:** *SMAD2* and *SMAD3* Depletion Affect the Transcription of Different Genes Involved in Cell Invasion.

	SMAD2 sh		SMAD3 sh	
Gene	Fold Regulation	*p*-Value	Fold Regulation	*p*-Value
**SMAD3 regulated genes**				
*RHOB*	1.54	ns	1.58	0.002
*ITGB2*	−1.28	ns	−1.80	0.013
*VIM*	−1.25	0.009	−1.53	0.001
**SMAD2 and SMAD3 regulated genes**				
*CSF1*	1.81	0.002	2.91	<0.001
*ARF6*	−1.55	0.011	−1.52	0.018

Only genes with a fold regulation value > 1.5 or <−1.5 and a *p*-value < 0.05 are presented. *n* = 4 (2 replicates × 2 independent experiments).

## Data Availability

The PCR array data presented in this study are available in Supplementary Table S1. Publicly available RNAseq datasets were analyzed in this study. This data can be found here: Gene Expression and Overall Survival Data Breast Invasive Carcinoma (TCGA, PanCancer Atlas) https://www.cbioportal.org/study/summary?id=brca_tcga_pan_can_atlas_2018, accessed on 7 January 2020; Lung Adenocarcinoma (TCGA, PanCancer Atlas) https://www.cbioportal.org/study/summary?id=luad_tcga_pan_can_atlas_2018, accessed on 7 January 2020; Sarcoma (TCGA, Firehose Legacy, accessed on 7 January 2020) https://www.cbioportal.org/study/summary?id=sarc_tcga, accessed on 7 January 2020. Metastasis or recurrence-free survival data *(*http://bioinformatica.mty.itesm.mx:8080/Biomatec/SurvivaX.jsp, accessed on 8 July 2020) Van’t Veer - Van De Vijver Nature 2002 (breast), Lee Son Lung GSE8894 (lung).

## Supplementary Materials

The following supporting information can be downloaded at: https://www.mdpi.com/article/10.3390/cancers14112751/s1, Figure S1: pSMAD2 Immunostaining in Tumor Xenografts. Figure S2: Effect of Hypoxia on SARA/SMAD Interaction and SARA mRNA Expression. Figure S3: Validation of the Efficacy of HDAC6 Inhibitors on Tubulin Acetylation. Figure S4: Effect of ITGB2 Blocking Antibodies on Invadopodia Production. Figure S5: Western Blotting Validation of Rescue Expression of VIM and ITGB2 in SMAD3-Depleted Cells. Table S1: PCR Array Complete Normalized Results. File S1: original Western Blot figures.

## References

[1] Massagué J. (2008). TGFbeta in Cancer. Cell.

[2] Seoane J., Gomis R.R. (2017). TGF-β Family Signaling in Tumor Suppression and Cancer Progression. Cold Spring Harb Perspect. Biol..

[3] Friedman E., Gold L.I., Klimstra D., Zeng Z.S., Winawer S., Cohen A. (1995). High Levels of Transforming Growth Factor Beta 1 Correlate with Disease Progression in Human Colon Cancer. Cancer Epidemiol. Biomark. Prev..

[4] Tsushima H., Kawata S., Tamura S., Ito N., Shirai Y., Kiso S., Imai Y., Shimomukai H., Nomura Y., Matsuda Y. (1996). High Levels of Transforming Growth Factor Beta 1 in Patients with Colorectal Cancer: Association with Disease Progression. Gastroenterology.

[5] Walker R.A., Dearing S.J. (1992). Transforming Growth Factor Beta 1 in Ductal Carcinoma in Situ and Invasive Carcinomas of the Breast. Eur. J. Cancer.

[6] Wikström P., Stattin P., Franck-Lissbrant I., Damber J.E., Bergh A. (1998). Transforming Growth Factor Beta1 Is Associated with Angiogenesis, Metastasis, and Poor Clinical Outcome in Prostate Cancer. Prostate.

[7] Buck M.B., Fritz P., Dippon J., Zugmaier G., Knabbe C. (2004). Prognostic Significance of Transforming Growth Factor Beta Receptor II in Estrogen Receptor-Negative Breast Cancer Patients. Clin. Cancer Res..

[8] Dalal B.I., Keown P.A., Greenberg A.H. (1993). Immunocytochemical Localization of Secreted Transforming Growth Factor-Beta 1 to the Advancing Edges of Primary Tumors and to Lymph Node Metastases of Human Mammary Carcinoma. Am. J. Pathol..

[9] Padua D., Zhang X.H.-F., Wang Q., Nadal C., Gerald W.L., Gomis R.R., Massagué J. (2008). TGFbeta Primes Breast Tumors for Lung Metastasis Seeding through Angiopoietin-like 4. Cell.

[10] Ciardiello D., Elez E., Tabernero J., Seoane J. (2020). Clinical Development of Therapies Targeting TGFβ: Current Knowledge and Future Perspectives. Ann. Oncol..

[11] Teixeira A.F., ten Dijke P., Zhu H.-J. (2020). On-Target Anti-TGF-β Therapies Are Not Succeeding in Clinical Cancer Treatments: What Are Remaining Challenges?. Front. Cell Dev. Biol..

[12] Brandes A.A., Carpentier A.F., Kesari S., Sepulveda-Sanchez J.M., Wheeler H.R., Chinot O., Cher L., Steinbach J.P., Capper D., Specenier P. (2016). A Phase II Randomized Study of Galunisertib Monotherapy or Galunisertib plus Lomustine Compared with Lomustine Monotherapy in Patients with Recurrent Glioblastoma. Neuro-Oncology.

[13] Akhurst R.J., Derynck R. (2001). TGF-Beta Signaling in Cancer—A Double-Edged Sword. Trends Cell Biol..

[14] Lebrun J.-J. (2012). The Dual Role of TGFβ in Human Cancer: From Tumor Suppression to Cancer Metastasis. ISRN Mol. Biol..

[15] Hill C.S. (2016). Transcriptional Control by the SMADs. Cold Spring Harb. Perspect. Biol..

[16] Tian F., Byfield S.D., Parks W.T., Stuelten C.H., Nemani D., Zhang Y.E., Roberts A.B. (2004). Smad-Binding Defective Mutant of Transforming Growth Factor Beta Type I Receptor Enhances Tumorigenesis but Suppresses Metastasis of Breast Cancer Cell Lines. Cancer Res..

[17] Singha P.K., Pandeswara S., Geng H., Lan R., Venkatachalam M.A., Dobi A., Srivastava S., Saikumar P. (2019). Increased Smad3 and Reduced Smad2 Levels Mediate the Functional Switch of TGF-β from Growth Suppressor to Growth and Metastasis Promoter through TMEPAI/PMEPA1 in Triple Negative Breast Cancer. Genes Cancer.

[18] Hoot K.E., Lighthall J., Han G., Lu S.-L., Li A., Ju W., Kulesz-Martin M., Bottinger E., Wang X.-J. (2008). Keratinocyte-Specific Smad2 Ablation Results in Increased Epithelial-Mesenchymal Transition during Skin Cancer Formation and Progression. J. Clin. Investig..

[19] Chen Y., Xing P., Chen Y., Zou L., Zhang Y., Li F., Lu X. (2014). High P-Smad2 Expression in Stromal Fibroblasts Predicts Poor Survival in Patients with Clinical Stage I to IIIA Non-Small Cell Lung Cancer. World J. Surg. Oncol..

[20] Cho S.Y., Ha S.Y., Huang S.-M., Kim J.H., Kang M.S., Yoo H.-Y., Kim H., Park C.-K., Um S.-H., Kim K.-H. (2014). The Prognostic Significance of Smad3, Smad4, Smad3 Phosphoisoform Expression in Esophageal Squamous Cell Carcinoma. Med. Oncol..

[21] Sferra R., Pompili S., Festuccia C., Marampon F., Gravina G.L., Ventura L., Di Cesare E., Cicchinelli S., Gaudio E., Vetuschi A. (2017). The Possible Prognostic Role of Histone Deacetylase and Transforming Growth Factor β/Smad Signaling in High Grade Gliomas Treated by Radio-Chemotherapy: A Preliminary Immunohistochemical Study. Eur. J. Histochem..

[22] Petersen M., Pardali E., van der Horst G., Cheung H., van den Hoogen C., van der Pluijm G., Ten Dijke P. (2010). Smad2 and Smad3 Have Opposing Roles in Breast Cancer Bone Metastasis by Differentially Affecting Tumor Angiogenesis. Oncogene.

[23] Ungefroren H., Groth S., Sebens S., Lehnert H., Gieseler F., Fändrich F. (2011). Differential Roles of Smad2 and Smad3 in the Regulation of TGF-Β1-Mediated Growth Inhibition and Cell Migration in Pancreatic Ductal Adenocarcinoma Cells: Control by Rac1. Mol. Cancer.

[24] Mingyuan X., Qianqian P., Shengquan X., Chenyi Y., Rui L., Yichen S., Jinghong X. (2017). Hypoxia-Inducible Factor-1α Activates Transforming Growth Factor-Β1/Smad Signaling and Increases Collagen Deposition in Dermal Fibroblasts. Oncotarget.

[25] Mori Y., Chen S.J., Varga J. (2000). Modulation of Endogenous Smad Expression in Normal Skin Fibroblasts by Transforming Growth Factor-Beta. Exp. Cell Res..

[26] Dong C., Li Z., Alvarez R., Feng X.H., Goldschmidt-Clermont P.J. (2000). Microtubule Binding to Smads May Regulate TGF Beta Activity. Mol. Cell.

[27] Watanabe Y., Itoh S., Goto T., Ohnishi E., Inamitsu M., Itoh F., Satoh K., Wiercinska E., Yang W., Shi L. (2010). TMEPAI, a Transmembrane TGF-Beta-Inducible Protein, Sequesters Smad Proteins from Active Participation in TGF-Beta Signaling. Mol. Cell.

[28] Hsieh C.-H., Shyu W.-C., Chiang C.-Y., Kuo J.-W., Shen W.-C., Liu R.-S. (2011). NADPH Oxidase Subunit 4-Mediated Reactive Oxygen Species Contribute to Cycling Hypoxia-Promoted Tumor Progression in Glioblastoma Multiforme. PLoS ONE.

[29] Rofstad E.K., Gaustad J.-V., Egeland T.A.M., Mathiesen B., Galappathi K. (2010). Tumors Exposed to Acute Cyclic Hypoxic Stress Show Enhanced Angiogenesis, Perfusion and Metastatic Dissemination. Int. J. Cancer.

[30] Lu Y., Hu J., Sun W., Duan X., Chen X. (2015). Hypoxia-Mediated Immune Evasion of Pancreatic Carcinoma Cells. Mol. Med. Rep..

[31] Cairns R.A., Hill R.P. (2004). Acute Hypoxia Enhances Spontaneous Lymph Node Metastasis in an Orthotopic Murine Model of Human Cervical Carcinoma. Cancer Res..

[32] Hung S.-P., Yang M.-H., Tseng K.-F., Lee O.K. (2013). Hypoxia-Induced Secretion of TGF-Β1 in Mesenchymal Stem Cell Promotes Breast Cancer Cell Progression. Cell Transplant..

[33] Arsenault D., Lucien F., Dubois C.M. (2012). Hypoxia Enhances Cancer Cell Invasion through Relocalization of the Proprotein Convertase Furin from the Trans-Golgi Network to the Cell Surface. J. Cell. Physiol..

[34] McMahon S., Grondin F., McDonald P.P., Richard D.E., Dubois C.M. (2005). Hypoxia-Enhanced Expression of the Proprotein Convertase Furin Is Mediated by Hypoxia-Inducible Factor-1: Impact on the Bioactivation of Proproteins. J. Biol. Chem..

[35] Cui W., Zhou J., Dehne N., Brüne B. (2015). Hypoxia Induces Calpain Activity and Degrades SMAD2 to Attenuate TGFβ Signaling in Macrophages. Cell Biosci..

[36] Zhang H., Akman H.O., Smith E.L.P., Zhao J., Murphy-Ullrich J.E., Batuman O.A. (2003). Cellular Response to Hypoxia Involves Signaling via Smad Proteins. Blood.

[37] Arsenault D., Brochu-Gaudreau K., Charbonneau M., Dubois C.M. (2013). HDAC6 Deacetylase Activity Is Required for Hypoxia-Induced Invadopodia Formation and Cell Invasion. PLoS ONE.

[38] Abdollah S., Macías-Silva M., Tsukazaki T., Hayashi H., Attisano L., Wrana J.L. (1997). TbetaRI Phosphorylation of Smad2 on Ser465 and Ser467 Is Required for Smad2-Smad4 Complex Formation and Signaling. J. Biol. Chem..

[39] Lucien F., Pelletier P.-P., Lavoie R.R., Lacroix J.-M., Roy S., Parent J.-L., Arsenault D., Harper K., Dubois C.M. (2017). Hypoxia-Induced Mobilization of NHE6 to the Plasma Membrane Triggers Endosome Hyperacidification and Chemoresistance. Nat. Commun..

[40] Zijlstra A., Mellor R., Panzarella G., Aimes R.T., Hooper J.D., Marchenko N.D., Quigley J.P. (2002). A Quantitative Analysis of Rate-Limiting Steps in the Metastatic Cascade Using Human-Specific Real-Time Polymerase Chain Reaction. Cancer Res..

[41] Baldassarre M., Ayala I., Beznoussenko G., Giacchetti G., Machesky L.M., Luini A., Buccione R. (2006). Actin Dynamics at Sites of Extracellular Matrix Degradation. Eur. J. Cell Biol..

[42] Wu G., Chen Y.G., Ozdamar B., Gyuricza C.A., Chong P.A., Wrana J.L., Massagué J., Shi Y. (2000). Structural Basis of Smad2 Recognition by the Smad Anchor for Receptor Activation. Science.

[43] Harper K., Arsenault D., Boulay-Jean S., Lauzier A., Lucien F., Dubois C.M. (2010). Autotaxin Promotes Cancer Invasion via the Lysophosphatidic Acid Receptor 4: Participation of the Cyclic AMP/EPAC/Rac1 Signaling Pathway in Invadopodia Formation. Cancer Res..

[44] Liu J., Lichtenberg T., Hoadley K.A., Poisson L.M., Lazar A.J., Cherniack A.D., Kovatich A.J., Benz C.C., Levine D.A., Lee A.V. (2018). An Integrated TCGA Pan-Cancer Clinical Data Resource to Drive High-Quality Survival Outcome Analytics. Cell.

[45] Buffa F.M., Harris A.L., West C.M., Miller C.J. (2010). Large Meta-Analysis of Multiple Cancers Reveals a Common, Compact and Highly Prognostic Hypoxia Metagene. Br. J. Cancer.

[46] Chen Y.-L., Zhang Y., Wang J., Chen N., Fang W., Zhong J., Liu Y., Qin R., Yu X., Sun Z. (2019). A 17 Gene Panel for Non-Small-Cell Lung Cancer Prognosis Identified through Integrative Epigenomic-Transcriptomic Analyses of Hypoxia-Induced Epithelial-Mesenchymal Transition. Mol. Oncol..

[47] Yang L., Forker L., Irlam J.J., Pillay N., Choudhury A., West C.M.L. (2018). Validation of a Hypoxia Related Gene Signature in Multiple Soft Tissue Sarcoma Cohorts. Oncotarget.

[48] Chibon F., Lagarde P., Salas S., Pérot G., Brouste V., Tirode F., Lucchesi C., de Reynies A., Kauffmann A., Bui B. (2010). Validated Prediction of Clinical Outcome in Sarcomas and Multiple Types of Cancer on the Basis of a Gene Expression Signature Related to Genome Complexity. Nat. Med..

[49] Lee E.-S., Son D.-S., Kim S.-H., Lee J., Jo J., Han J., Kim H., Lee H.J., Choi H.Y., Jung Y. (2008). Prediction of Recurrence-Free Survival in Postoperative Non-Small Cell Lung Cancer Patients by Using an Integrated Model of Clinical Information and Gene Expression. Clin. Cancer Res..

[50] Van’t Veer L.J., Dai H., van de Vijver M.J., He Y.D., Hart A.A.M., Mao M., Peterse H.L., van der Kooy K., Marton M.J., Witteveen A.T. (2002). Gene Expression Profiling Predicts Clinical Outcome of Breast Cancer. Nature.

[51] Aguirre-Gamboa R., Gomez-Rueda H., Martínez-Ledesma E., Martínez-Torteya A., Chacolla-Huaringa R., Rodriguez-Barrientos A., Tamez-Peña J.G., Treviño V. (2013). SurvExpress: An Online Biomarker Validation Tool and Database for Cancer Gene Expression Data Using Survival Analysis. PLoS ONE.

[52] Sugiyama Y., Kakoi K., Kimura A., Takada I., Kashiwagi I., Wakabayashi Y., Morita R., Nomura M., Yoshimura A. (2012). Smad2 and Smad3 Are Redundantly Essential for the Suppression of INOS Synthesis in Macrophages by Regulating IRF3 and STAT1 Pathways. Int. Immunol..

[53] Takimoto T., Wakabayashi Y., Sekiya T., Inoue N., Morita R., Ichiyama K., Takahashi R., Asakawa M., Muto G., Mori T. (2010). Smad2 and Smad3 Are Redundantly Essential for the TGF-Beta-Mediated Regulation of Regulatory T Plasticity and Th1 Development. J. Immunol..

[54] Brown K.A., Pietenpol J.A., Moses H.L. (2007). A Tale of Two Proteins: Differential Roles and Regulation of Smad2 and Smad3 in TGF-Beta Signaling. J. Cell. Biochem..

[55] Piek E., Ju W.J., Heyer J., Escalante-Alcalde D., Stewart C.L., Weinstein M., Deng C., Kucherlapati R., Bottinger E.P., Roberts A.B. (2001). Functional Characterization of Transforming Growth Factor Beta Signaling in Smad2- and Smad3-Deficient Fibroblasts. J. Biol. Chem..

[56] Zhang L.-H., Tian B., Diao L.-R., Xiong Y.-Y., Tian S.-F., Zhang B.-H., Li W.-M., Ren H., Li Y., Ji J.-F. (2006). Dominant Expression of 85-KDa Form of Cortactin in Colorectal Cancer. J. Cancer Res. Clin. Oncol..

[57] Hirooka S., Akashi T., Ando N., Suzuki Y., Ishida N., Kurata M., Takizawa T., Kayamori K., Sakamoto K., Fujiwara N. (2011). Localization of the Invadopodia-Related Proteins Actinin-1 and Cortactin to Matrix-Contact-Side Cytoplasm of Cancer Cells in Surgically Resected Lung Adenocarcinomas. Pathobiology.

[58] Leong H.S., Robertson A.E., Stoletov K., Leith S.J., Chin C.A., Chien A.E., Hague M.N., Ablack A., Carmine-Simmen K., McPherson V.A. (2014). Invadopodia Are Required for Cancer Cell Extravasation and Are a Therapeutic Target for Metastasis. Cell Rep..

[59] Harper K., Yatsyna A., Charbonneau M., Brochu-Gaudreau K., Perreault A., Jeldres C., McDonald P.P., Dubois C.M. (2021). The Chicken Chorioallantoic Membrane Tumor Assay as a Relevant In Vivo Model to Study the Impact of Hypoxia on Tumor Progression and Metastasis. Cancers.

[60] Tsukazaki T., Chiang T.A., Davison A.F., Attisano L., Wrana J.L. (1998). SARA, a FYVE Domain Protein That Recruits Smad2 to the TGFbeta Receptor. Cell.

[61] Runyan C.E., Schnaper H.W., Poncelet A.-C. (2005). The Role of Internalization in Transforming Growth Factor Beta1-Induced Smad2 Association with Smad Anchor for Receptor Activation (SARA) and Smad2-Dependent Signaling in Human Mesangial Cells. J. Biol. Chem..

[62] Tang W., Ling G., Sun L., Liu F.-Y. (2010). Smad Anchor for Receptor Activation (SARA) in TGF-Beta Signaling. Front. Biosci. (Elite Ed).

[63] Tang W., Ling G., Sun L., Zhang K., Zhu X., Zhou X., Liu F. (2015). Smad Anchor for Receptor Activation Regulates High Glucose-Induced EMT via Modulation of Smad2 and Smad3 Activities in Renal Tubular Epithelial Cells. Nephron.

[64] Goto D., Nakajima H., Mori Y., Kurasawa K., Kitamura N., Iwamoto I. (2001). Interaction between Smad Anchor for Receptor Activation and Smad3 Is Not Essential for TGF-Beta/Smad3-Mediated Signaling. Biochem. Biophys. Res. Commun..

[65] Henke R.M., Dastidar R.G., Shah A., Cadinu D., Yao X., Hooda J., Zhang L. (2011). Hypoxia Elicits Broad and Systematic Changes in Protein Subcellular Localization. Am. J. Physiol. Cell Physiol..

[66] Bensellam M., Maxwell E.L., Chan J.Y., Luzuriaga J., West P.K., Jonas J.-C., Gunton J.E., Laybutt D.R. (2016). Hypoxia Reduces ER-to-Golgi Protein Trafficking and Increases Cell Death by Inhibiting the Adaptive Unfolded Protein Response in Mouse Beta Cells. Diabetologia.

[67] Di Guglielmo G.M., Le Roy C., Goodfellow A.F., Wrana J.L. (2003). Distinct Endocytic Pathways Regulate TGF-Beta Receptor Signalling and Turnover. Nat. Cell Biol..

[68] Hayes S., Chawla A., Corvera S. (2002). TGF Beta Receptor Internalization into EEA1-Enriched Early Endosomes: Role in Signaling to Smad2. J. Cell Biol..

[69] Penheiter S.G., Mitchell H., Garamszegi N., Edens M., Doré J.J.E., Leof E.B. (2002). Internalization-Dependent and -Independent Requirements for Transforming Growth Factor Beta Receptor Signaling via the Smad Pathway. Mol. Cell. Biol..

[70] Chalmers K.A., Love S. (2007). Neurofibrillary Tangles May Interfere with Smad 2/3 Signaling in Neurons. J. Neuropathol. Exp. Neurol..

[71] Xu L., Liu N., Gu H., Wang H., Shi Y., Ma X., Ma S., Ni J., Tao M., Qiu A. (2017). Histone Deacetylase 6 Inhibition Counteracts the Epithelial-Mesenchymal Transition of Peritoneal Mesothelial Cells and Prevents Peritoneal Fibrosis. Oncotarget.

[72] Melisi D., Garcia-Carbonero R., Macarulla T., Pezet D., Deplanque G., Fuchs M., Trojan J., Oettle H., Kozloff M., Cleverly A. (2018). Galunisertib plus Gemcitabine vs. Gemcitabine for First-Line Treatment of Patients with Unresectable Pancreatic Cancer. Br. J. Cancer.

[73] Faivre S., Santoro A., Kelley R.K., Gane E., Costentin C.E., Gueorguieva I., Smith C., Cleverly A., Lahn M.M., Raymond E. (2019). Novel Transforming Growth Factor Beta Receptor I Kinase Inhibitor Galunisertib (LY2157299) in Advanced Hepatocellular Carcinoma. Liver Int..

[74] Harris B.H.L., Barberis A., West C.M.L., Buffa F.M. (2015). Gene Expression Signatures as Biomarkers of Tumour Hypoxia. Clin. Oncol. (R. Coll. Radiol.).

[75] Tannehill-Gregg S.H., Kusewitt D.F., Rosol T.J., Weinstein M. (2004). The Roles of Smad2 and Smad3 in the Development of Chemically Induced Skin Tumors in Mice. Vet. Pathol..

[76] Xie W., Mertens J.C., Reiss D.J., Rimm D.L., Camp R.L., Haffty B.G., Reiss M. (2002). Alterations of Smad Signaling in Human Breast Carcinoma Are Associated with Poor Outcome: A Tissue Microarray Study. Cancer Res..

[77] Tian F., DaCosta Byfield S., Parks W.T., Yoo S., Felici A., Tang B., Piek E., Wakefield L.M., Roberts A.B. (2003). Reduction in Smad2/3 Signaling Enhances Tumorigenesis but Suppresses Metastasis of Breast Cancer Cell Lines. Cancer Res..

[78] Liu N., Qi D., Jiang J., Zhang J., Yu C. (2020). Expression Pattern of P-Smad2/Smad4 as a Predictor of Survival in Invasive Breast Ductal Carcinoma. Oncol. Lett..

[79] Wu Y., Li Q., Zhou X., Yu J., Mu Y., Munker S., Xu C., Shen Z., Müllenbach R., Liu Y. (2012). Decreased Levels of Active SMAD2 Correlate with Poor Prognosis in Gastric Cancer. PLoS ONE.

[80] Zhang H.-W., Guo Y., Sun L., Ni F., Xu K. (2021). Prognostic Value of Small Mother against Decapentaplegic Expression in Human Gastric Cancer. Bioengineered.

[81] Itoh F., Divecha N., Brocks L., Oomen L., Janssen H., Calafat J., Itoh S., ten Dijke P. (2002). The FYVE Domain in Smad Anchor for Receptor Activation (SARA) Is Sufficient for Localization of SARA in Early Endosomes and Regulates TGF-Beta/Smad Signalling. Genes Cells.

[82] He J., Vora M., Haney R.M., Filonov G.S., Musselman C.A., Burd C.G., Kutateladze A.G., Verkhusha V.V., Stahelin R.V., Kutateladze T.G. (2009). Membrane Insertion of the FYVE Domain Is Modulated by PH. Proteins.

[83] Lucien F., Harper K., Pelletier P.-P., Volkov L., Dubois C.M. (2014). Simultaneous pH Measurement in Endocytic and Cytosolic Compartments in Living Cells Using Confocal Microscopy. J. Vis. Exp..

[84] Panopoulou E., Gillooly D.J., Wrana J.L., Zerial M., Stenmark H., Murphy C., Fotsis T. (2002). Early Endosomal Regulation of Smad-Dependent Signaling in Endothelial Cells. J. Biol. Chem..

[85] Bakkebø M., Huse K., Hilden V.I., Forfang L., Myklebust J.H., Smeland E.B., Oksvold M.P. (2012). SARA Is Dispensable for Functional TGF-β Signaling. FEBS Lett..

[86] Rozés-Salvador V., Wilson C., Olmos C., Gonzalez-Billault C., Conde C. (2020). Fine-Tuning the TGFβ Signaling Pathway by SARA During Neuronal Development. Front. Cell Dev. Biol..

[87] Huang C., Du R., Zhang P., Meng H., Jia H., Song Y., Li M., Zhang Y., Sun S. (2011). Expression, Purification, and Functional Characterization of Recombinant PTD-SARA. Acta Biochim. Biophys. Sin..

[88] Zhao B.M., Hoffmann F.M. (2006). Inhibition of Transforming Growth Factor-Beta1-Induced Signaling and Epithelial-to-Mesenchymal Transition by the Smad-Binding Peptide Aptamer Trx-SARA. Mol. Biol. Cell.

[89] Richardson A.M., Havel L.S., Koyen A.E., Konen J.M., Shupe J., Wiles W.G., Martin W.D., Grossniklaus H.E., Sica G., Gilbert-Ross M. (2018). Vimentin Is Required for Lung Adenocarcinoma Metastasis via Heterotypic Tumor Cell-Cancer-Associated Fibroblast Interactions during Collective Invasion. Clin. Cancer Res..

[90] Liu S., Liu L., Ye W., Ye D., Wang T., Guo W., Liao Y., Xu D., Song H., Zhang L. (2016). High Vimentin Expression Associated with Lymph Node Metastasis and Predicated a Poor Prognosis in Oral Squamous Cell Carcinoma. Sci. Rep..

[91] Wang R., Du X., Zhi Y. (2020). Screening of Critical Genes Involved in Metastasis and Prognosis of High-Grade Serous Ovarian Cancer by Gene Expression Profile Data. J. Comput. Biol..

[92] Liu H., Dai X., Cao X., Yan H., Ji X., Zhang H., Shen S., Si Y., Zhang H., Chen J. (2018). PRDM4 Mediates YAP-Induced Cell Invasion by Activating Leukocyte-Specific Integrin Β2 Expression. EMBO Rep..

[93] Wang A., Chen M., Wang H., Huang J., Bao Y., Gan X., Liu B., Lu X., Wang L. (2019). Cell Adhesion-Related Molecules Play a Key Role in Renal Cancer Progression by Multinetwork Analysis. Biomed. Res. Int..

[94] Zhu S., He C., Deng S., Li X., Cui S., Zeng Z., Liu M., Zhao S., Chen J., Jin Y. (2016). MiR-548an, Transcriptionally Downregulated by HIF1α/HDAC1, Suppresses Tumorigenesis of Pancreatic Cancer by Targeting Vimentin Expression. Mol. Cancer Ther..

[95] Kong T., Eltzschig H.K., Karhausen J., Colgan S.P., Shelley C.S. (2004). Leukocyte Adhesion during Hypoxia Is Mediated by HIF-1-Dependent Induction of Beta2 Integrin Gene Expression. Proc. Natl. Acad. Sci. USA.

[96] Liu T., Guevara O.E., Warburton R.R., Hill N.S., Gaestel M., Kayyali U.S. (2010). Regulation of Vimentin Intermediate Filaments in Endothelial Cells by Hypoxia. Am. J. Physiol. Cell Physiol..

[97] Rogel M.R., Soni P.N., Troken J.R., Sitikov A., Trejo H.E., Ridge K.M. (2011). Vimentin Is Sufficient and Required for Wound Repair and Remodeling in Alveolar Epithelial Cells. FASEB J..

[98] Vasilaki E., Papadimitriou E., Tajadura V., Ridley A.J., Stournaras C., Kardassis D. (2010). Transcriptional Regulation of the Small GTPase RhoB Gene by TGF{beta}-Induced Signaling Pathways. FASEB J..

[99] Jang H.-R., Shin S.-B., Kim C.-H., Won J.-Y., Xu R., Kim D.-E., Yim H. (2021). PLK1/Vimentin Signaling Facilitates Immune Escape by Recruiting Smad2/3 to PD-L1 Promoter in Metastatic Lung Adenocarcinoma. Cell Death Differ..

[100] Yoshida K., Saito T., Kamida A., Matsumoto K., Saeki K., Mochizuki M., Sasaki N., Nakagawa T. (2013). Transforming Growth Factor-β Transiently Induces Vimentin Expression and Invasive Capacity in a Canine Mammary Gland Tumor Cell Line. Res. Vet. Sci..

[101] Yoon S.-O., Shin S., Mercurio A.M. (2005). Hypoxia Stimulates Carcinoma Invasion by Stabilizing Microtubules and Promoting the Rab11 Trafficking of the Alpha6beta4 Integrin. Cancer Res..

[102] Blaschke F., Stawowy P., Goetze S., Hintz O., Gräfe M., Kintscher U., Fleck E., Graf K. (2002). Hypoxia Activates Beta(1)-Integrin via ERK 1/2 and P38 MAP Kinase in Human Vascular Smooth Muscle Cells. Biochem. Biophys. Res. Commun..

[103] Bourseau-Guilmain E., Menard J.A., Lindqvist E., Indira Chandran V., Christianson H.C., Cerezo Magaña M., Lidfeldt J., Marko-Varga G., Welinder C., Belting M. (2016). Hypoxia Regulates Global Membrane Protein Endocytosis through Caveolin-1 in Cancer Cells. Nat. Commun..

[104] Kim B.-G., Malek E., Choi S.H., Ignatz-Hoover J.J., Driscoll J.J. (2021). Novel Therapies Emerging in Oncology to Target the TGF-β Pathway. J. Hematol. Oncol..

[105] Gómez-Gil V. (2021). Therapeutic Implications of TGFβ in Cancer Treatment: A Systematic Review. Cancers.

[106] Jinnin M., Ihn H., Tamaki K. (2006). Characterization of SIS3, a Novel Specific Inhibitor of Smad3, and Its Effect on Transforming Growth Factor-Beta1-Induced Extracellular Matrix Expression. Mol. Pharmacol..

[107] Kang J.-H., Jung M.-Y., Yin X., Andrianifahanana M., Hernandez D.M., Leof E.B. (2017). Cell-Penetrating Peptides Selectively Targeting SMAD3 Inhibit Profibrotic TGF-β Signaling. J. Clin. Investig..

[108] Liu X., Wang W., Hu H., Tang N., Zhang C., Liang W., Wang M. (2006). Smad3 Specific Inhibitor, Naringenin, Decreases the Expression of Extracellular Matrix Induced by TGF-Beta1 in Cultured Rat Hepatic Stellate Cells. Pharm. Res..

[109] Li J., Qu X., Yao J., Caruana G., Ricardo S.D., Yamamoto Y., Yamamoto H., Bertram J.F. (2010). Blockade of Endothelial-Mesenchymal Transition by a Smad3 Inhibitor Delays the Early Development of Streptozotocin-Induced Diabetic Nephropathy. Diabetes.

[110] Meng J., Qin Y., Chen J., Wei L., Huang X., Yu X., Lan H. (2020). Treatment of Hypertensive Heart Disease by Targeting Smad3 Signaling in Mice. Mol. Ther.-Methods Clin. Dev..

[111] Lian G.-Y., Wang Q.-M., Mak T.S.-K., Huang X.-R., Yu X.-Q., Lan H.-Y. (2021). Inhibition of Tumor Invasion and Metastasis by Targeting TGF-β-Smad-MMP2 Pathway with Asiatic Acid and Naringenin. Mol. Ther. Oncolytics.

