# Supplementary material for: Hypoxia Selectively Increases a SMAD3 Signaling Axis to Promote Cancer Cell Invasion
Source: Cancers (Basel). 2022 Jun 1;14(11):2751. doi: 10.3390/cancers14112751 (PMC9179584; doi:10.3390/cancers14112751)
Supplement: Supplementary file 1 [file cancers-14-02751-s001.zip › cancers-1754862-supplementary/Supp Material/SuppFig.pdf]

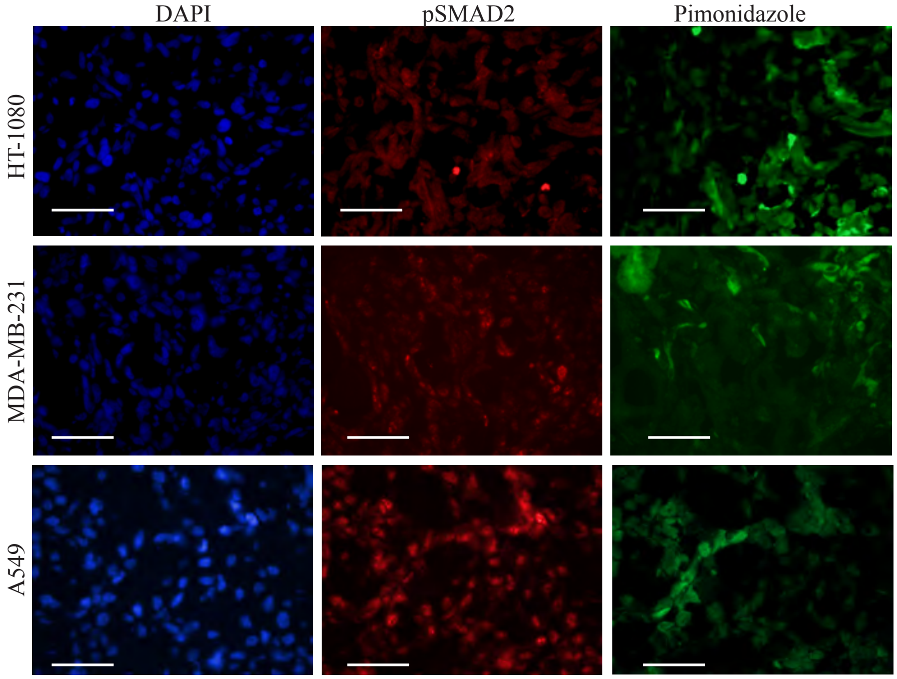

Figure S1: pSMAD2 Immunostaining in Tumor Xenografts.
Representative images of cryosections of HT1080, MDA-MB-231 and A549 xenograft tumors grown on chick embryo CAM, stained for nucleus (DAPI; blue), hypoxic regions (hypoxyprobe; red) and pSMAD2 (green). Scale bar = 50µm.

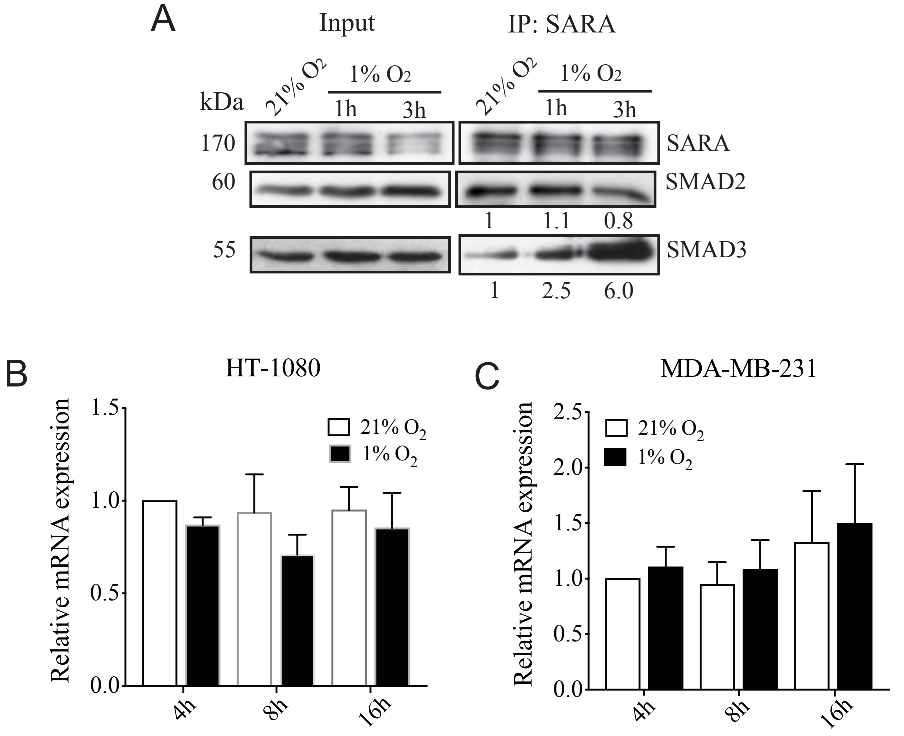

Figure S2. Effect of Hypoxia on SARA/SMAD Interaction and SARA mRNA Expression. A) Representative western blot images from co-immunoprecipitation of SARA in HT-1080 cells incubated under normoxic (21% O2) or hypoxic (1% O2) conditions (N=2-3). B-C) Relative SARA mRNA expression in HT-1080 (B) and MDA-MB-231 (C) cells incubated under normoxic (21% O2) or hypoxic (1% O2) conditions (N=4).

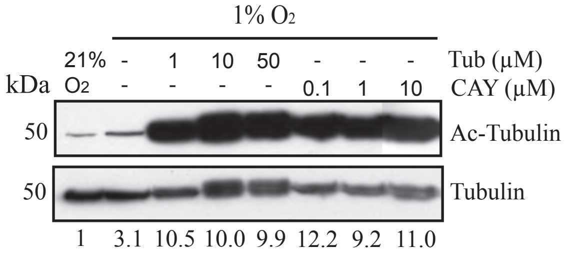

Figure S3: **Validation of the Efficacy of HDAC6 Inhibitors on Tubulin Acetylation**. HT-1080 cells were treated with the HDAC6 inhibitors tubacin (Tub) or CAY10603 (CAY), then incubated for 3h under hypoxic conditions. A representative immunoblot for acetylated tubulin (Ac-Tubulin), using tubulin as control, is presented (N=2).

Table S1: PCR Array Complete Normalized Results

sh SMAD2			sh SMAD3		
Gene	Fold (Log2)	p Value	Gene	Fold (Log2)	p Value
ACTN1	1,0512	0,650195	ACTN1	1,0282	0,755691
ACTN3	1,0512	0,650195	ACTN3	1,0282	0,755691
ACTN4	-1,601	0,001838	ACTN4	-1,4086	0,000934
ACTR2	-1,5846	0,005332	ACTR2	-1,1942	0,119284
ACTR3	-1,7358	0,005692	ACTR3	-1,1378	0,150733
AKT1	-1,6001	0,000079	AKT1	-1,1887	0,026884
ARF6	-1,5464	0,011389	ARF6	-1,5166	0,017677
ARHGDIA	-1,8705	0,000153	ARHGDIA	-1,1411	0,128329
ARHGEF7	-1,3619	0,086617	ARHGEF7	-1,0051	0,901162
BAIAP2	-1,0768	0,572282	BAIAP2	1,0382	0,96586
BCAR1	7,7734	0,644358	BCAR1	2,8157	0,800436
CAPN1	3,3695	0,257221	CAPN1	2,7556	0,340549
CAPN2	-1,6005	0,045627	CAPN2	-1,1983	0,294669
CAV1	-1,9155	0,02281	CAV1	-1,1182	0,654529
CDC42	-1,4844	0,000306	CDC42	-1,3276	0,001692
CFL1	-1,3818	0,008717	CFL1	-1,1571	0,121643
CRK	-1,4178	0,00247	CRK	-1,1308	0,139902
CSF1	1,8077	0,001682	CSF1	2,9101	0,000057
CTTN	-1,2056	0,689472	CTTN	-1,2386	0,250915
DIAPH1	-2,0755	0,000183	DIAPH1	-1,1467	0,088939
DPP4	1,0512	0,650195	DPP4	1,0282	0,755691
EGF	-1,4252	0,223434	EGF	1,4547	0,281117
EGFR	1,1665	0,276396	EGFR	-1,0034	0,995355
ENAH	-1,1566	0,096705	ENAH	1,1794	0,218943
EZR	-1,3214	0,030904	EZR	-1,1323	0,001338
FAP	1,0512	0,650195	FAP	1,0282	0,755691
FGF2	-1,0173	0,864536	FGF2	1,1111	0,123445
HGF	1,0512	0,650195	HGF	1,0282	0,755691
IGF1	1,0512	0,650195	IGF1	1,1077	0,466248
IGF1R	-1,3623	0,246047	IGF1R	1,5563	0,0561
ILK	-1,475	0,001832	ILK	-1,1302	0,157994
ITGA4	-1,3472	0,078309	ITGA4	-1,2799	0,162907
ITGB1	-1,1494	0,03276	ITGB1	-1,2263	0,015181
ITGB2	-1,2785	0,824238	ITGB2	-1,7993	0,012775
ITGB3	2,1815	0,205607	ITGB3	1,091	0,909822
LIMK1	1,1142	0,18354	LIMK1	1,4512	0,032482
MAPK1	-1,3538	0,232342	MAPK1	-1,1575	0,483864
MET	-1,1722	0,127515	MET	-4,3224	0,713818
MMP14	-1,5152	0,259784	MMP14	-1,1613	0,221749
MMP2	1,6245	0,011912	MMP2	1,1854	0,096688
MMP9	3,6357	0,161805	MMP9	4,6558	0,138464
MSN	-1,8227	0,000002	MSN	-1,4047	0,0006
MYH10	-1,4332	0,008434	MYH10	-1,0245	0,9826
MYH9	-1,399	0,018915	MYH9	-1,1415	0,085584
MYL9	-1,074	0,569954	MYL9	-1,374	0,000699
MYLK	1,0421	0,657063	MYLK	1,3067	0,097294
PAK1	-1,3507	0,266493	PAK1	-1,149	0,486884
PAK4	-1,4609	0,000372	PAK4	-1,263	0,005248
PFN1	-1,31	0,00256	PFN1	-1,361	0,000983
PIK3CA	-1,8204	0,00695	PIK3CA	-1,1945	0,092391
PLAUR	-1,1847	0,569853	PLAUR	-1,4437	0,001498

sh SMAD2			sh SMAD3		
**Gene**	**Fold (Log2)**	**p Value**	**Gene**	**Fold (Log2)**	**p Value**
PLCG1	-1,6619	0,011998	PLCG1	-1,4048	0,114628
PLD1	-1,5945	0,08953	PLD1	-1,0753	0,794592
PRKCA	-1,119	0,651282	PRKCA	-1,0273	0,867933
PTEN	-1,3044	0,016048	PTEN	-1,1469	0,231486
PTK2	-1,5374	0,154967	PTK2	-1,152	0,336985
PTK2B	1,3399	0,342445	PTK2B	-1,1323	0,574421
PTPN1	-1,7716	0,000921	PTPN1	-1,4411	0,022361
PXN	-1,4678	0,000469	PXN	-1,2314	0,009301
RAC1	-1,3389	0,049376	RAC1	-1,2343	0,092917
RAC2	-1,241	0,072	RAC2	1,0798	0,517279
RASA1	-1,9121	0,024709	RASA1	-1,2718	0,13716
RDX	-1,6191	0,159707	RDX	1,1605	0,430101
RHO	1,0512	0,650195	RHO	1,0282	0,755691
RHOA	-1,3772	0,0577	RHOA	-1,2803	0,062245
RHOB	1,5424	0,060448	RHOB	1,582	0,001676
RHOC	-1,5177	0,005159	RHOC	-1,4228	0,016014
RND3	1,0785	0,578103	RND3	-1,1158	0,346226
ROCK1	-1,4714	0,00795	ROCK1	-1,2128	0,08207
SH3PXD2A	-1,7536	0,062889	SH3PXD2A	-1,5416	0,152633
SRC	-1,392	0,04199	SRC	1,09	0,554541
STAT3	-1,4132	0,064154	STAT3	1,4046	0,002208
SVIL	-1,7959	0,067608	SVIL	-1,8811	0,050104
TGFB1	-1,4177	0,018502	TGFB1	-1,3438	0,043888
TIMP2	-1,4744	0,171787	TIMP2	1,3517	0,019804
TLN1	-1,3229	0,006406	TLN1	-1,327	0,018815
VASP	-2,0591	0,007324	VASP	-1,0992	0,518799
VCL	-2,0449	0,002523	VCL	-1,0169	0,856272
VEGFA	1,1079	0,212341	VEGFA	-1,3397	0,11335
VIM	-1,2501	0,009462	VIM	-1,5326	0,001167
WASF1	-1,0664	0,351211	WASF1	-3,1729	0,502118
WASF2	-1,3823	0,025879	WASF2	-1,22	0,07723
WASL	-1,4031	0,377272	WASL	1,0805	0,805697
WIPF1	-1,6813	0,099109	WIPF1	-1,6222	0,088615
B2M	1,0906	0,131185	B2M	1,2025	0,007214
RPLP0	-1,0754	0,13396	RPLP0	-1,1689	0,009126

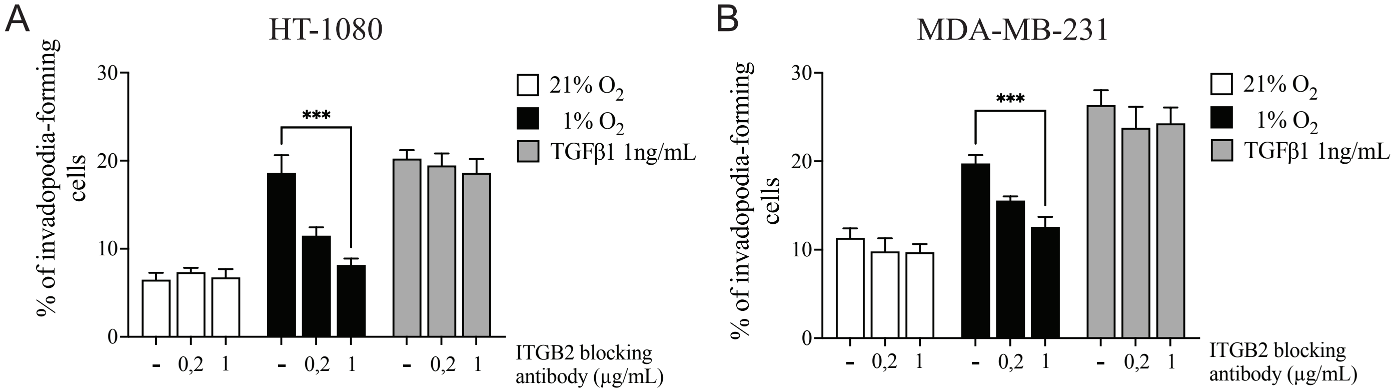

Figure S4: Effect of ITGB2 Blocking Antibody on Invadopodia Production.
HT-1080 (A) and MDA-MB-231 (B) cells were submitted to invadopodia assays under normoxic (21%), hypoxic (1% O2) or TGFβ1 supplemented conditions in the presence of ITGB2 blocking antibody or isotype control. (N=4) Results are presented as mean +/- SEM. *** $P < 0.001$.

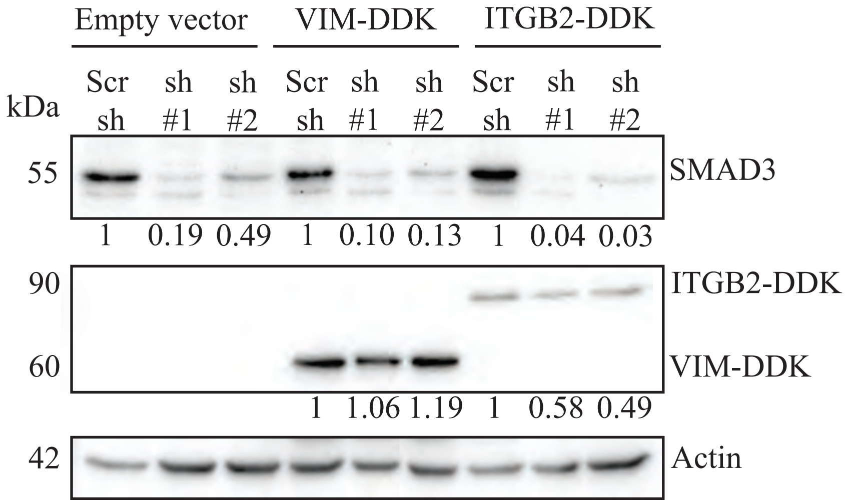

Figure S5. **Western Blotting Validation of Rescue Expression of VIM and ITGB2 in SMAD3-Depleted Cells.** HT1080 cells were immunoblotted for SMAD3, DDK (flag) and actin, as a loading control. Representative blots are presented (N=5).
