# Supplementary material for: Hypoxia Selectively Increases a SMAD3 Signaling Axis to Promote Cancer Cell Invasion
Source: Cancers (Basel). 2022 Jun 1;14(11):2751. doi: 10.3390/cancers14112751 (PMC9179584; doi:10.3390/cancers14112751)

Figure 3 - Original Blots

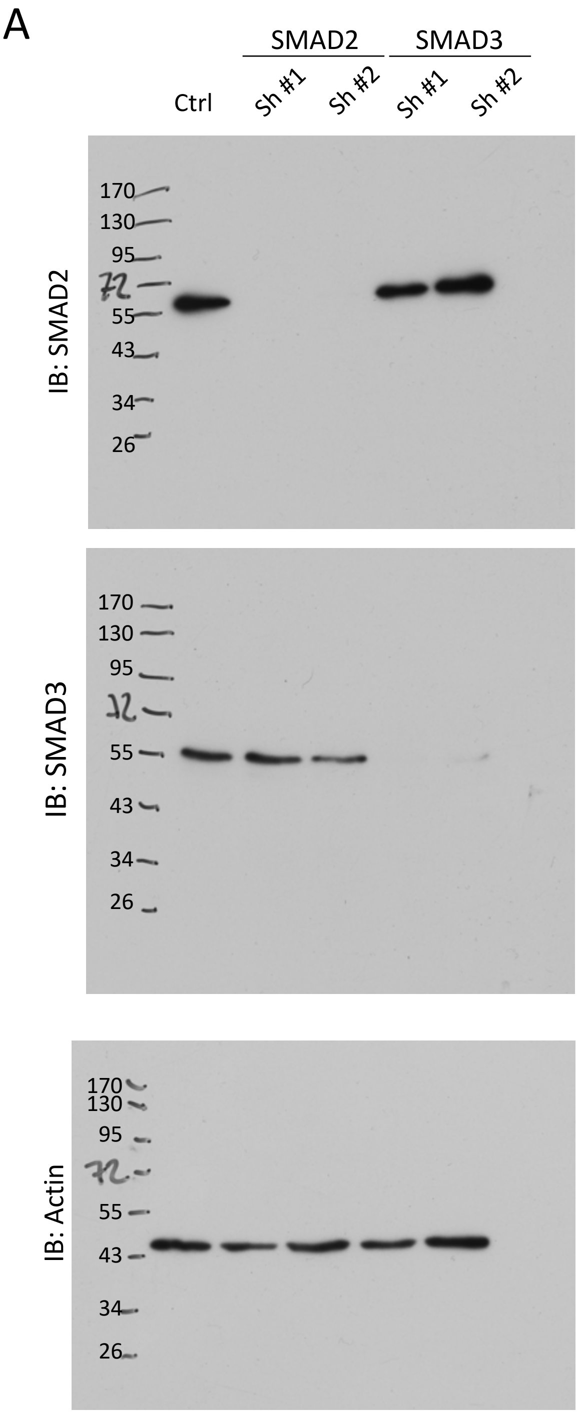

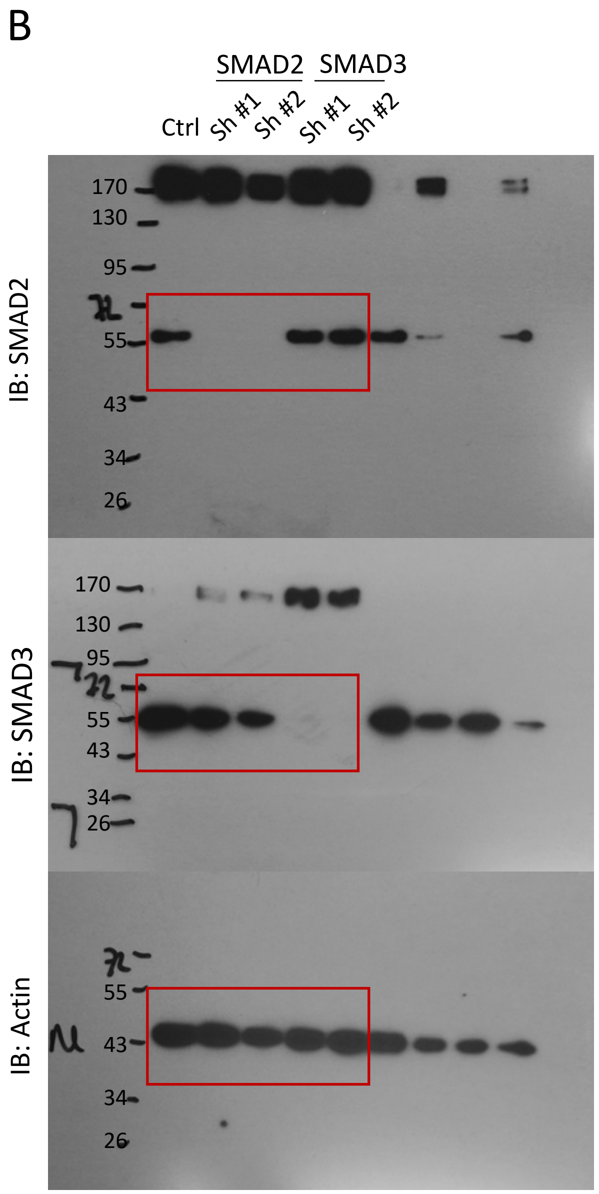

Figure 5 - Original Blots

A

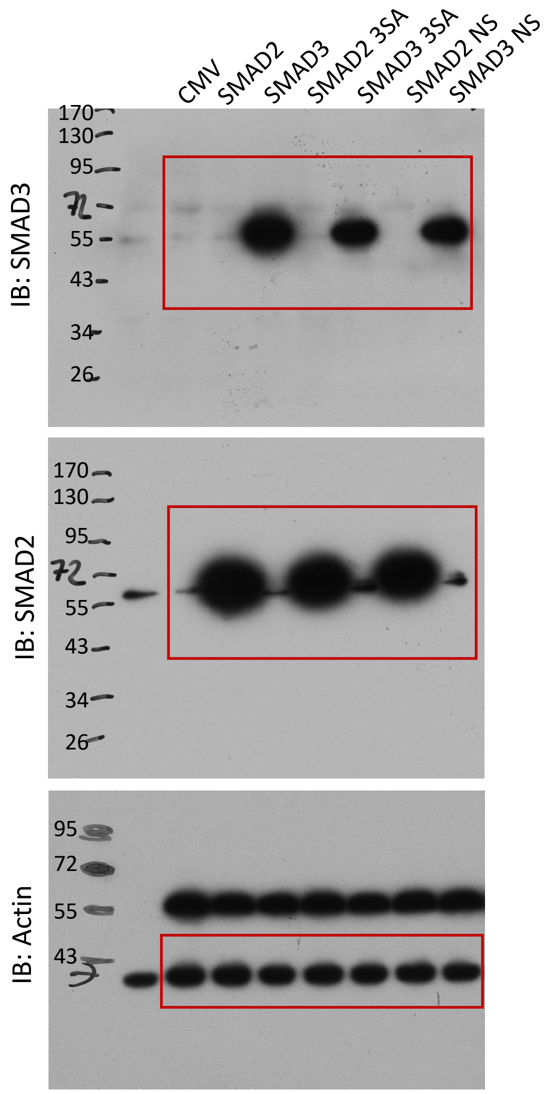

B

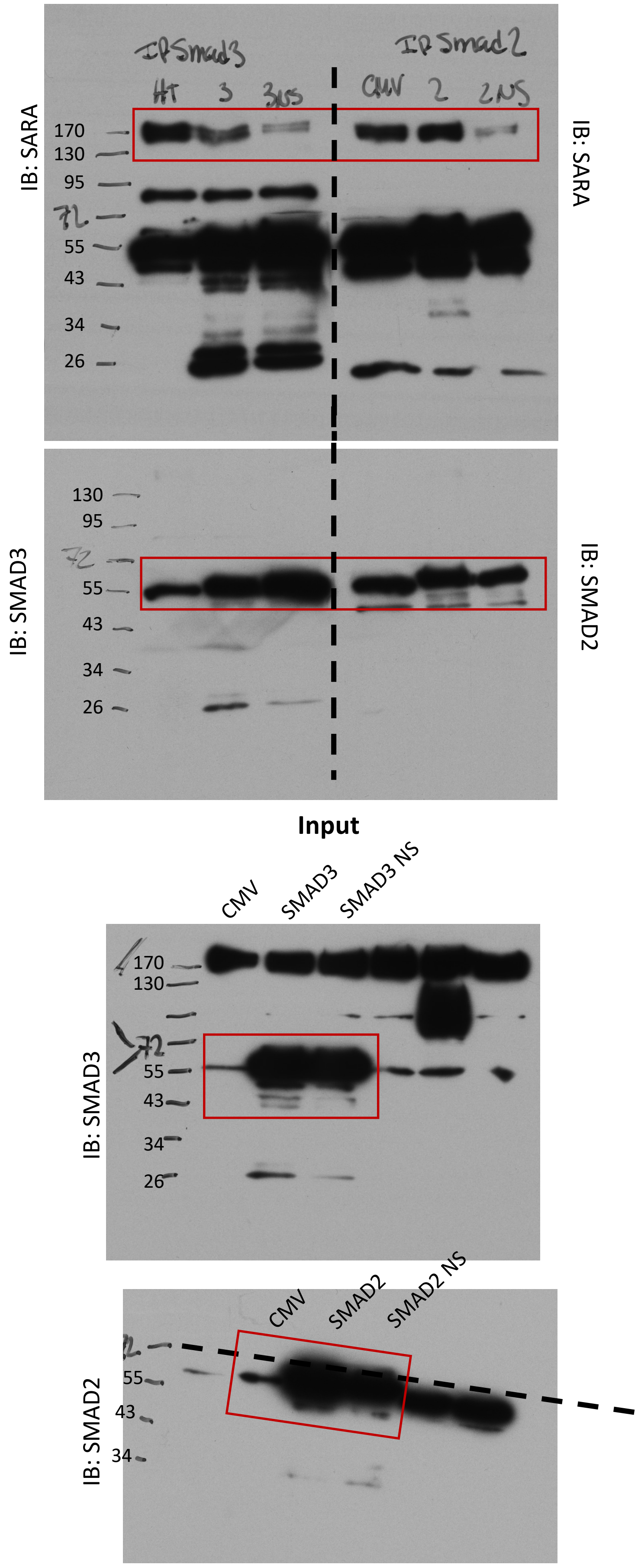

Figure 6 - Original Blots

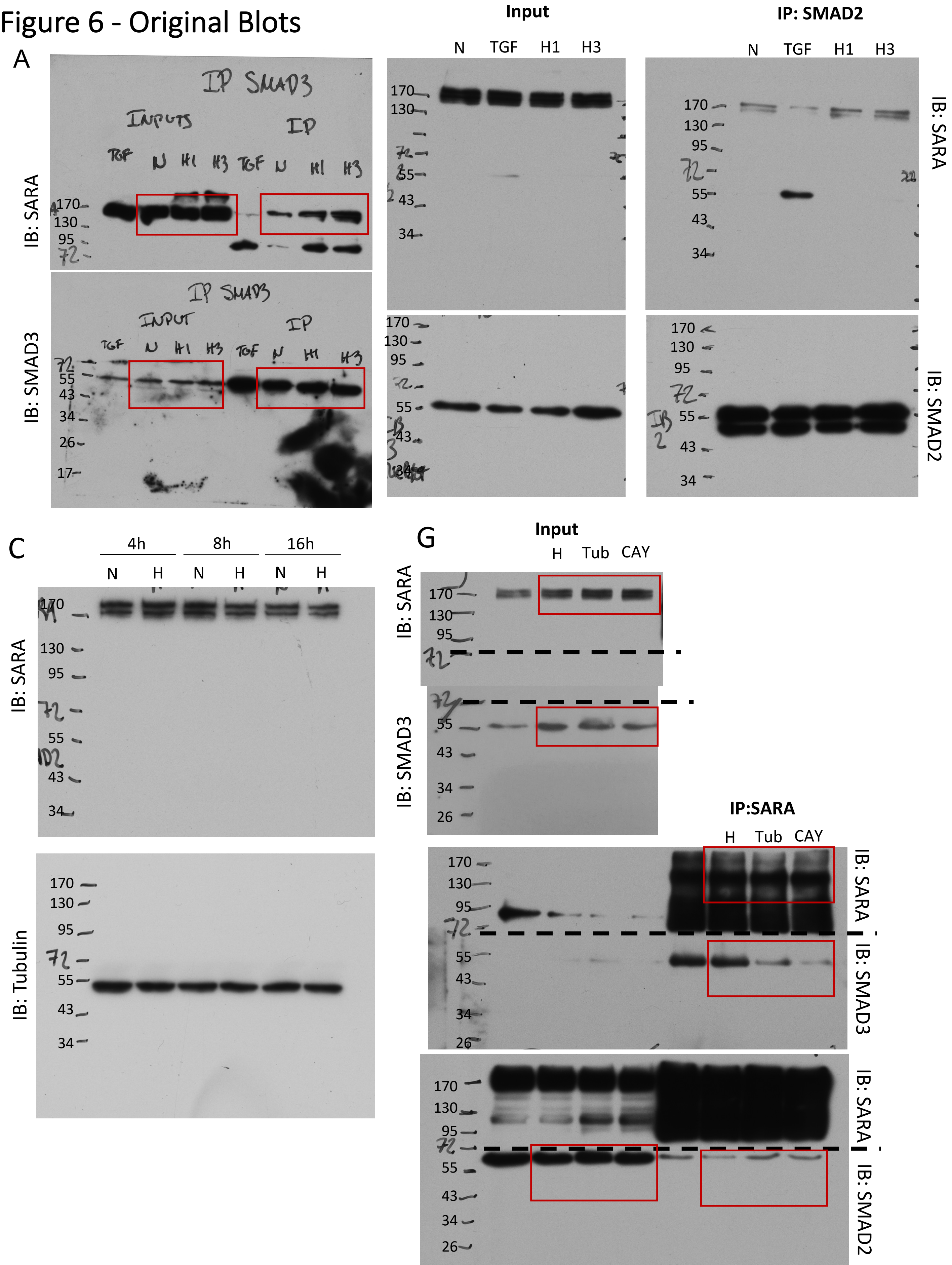

Figure S2 - Original blots

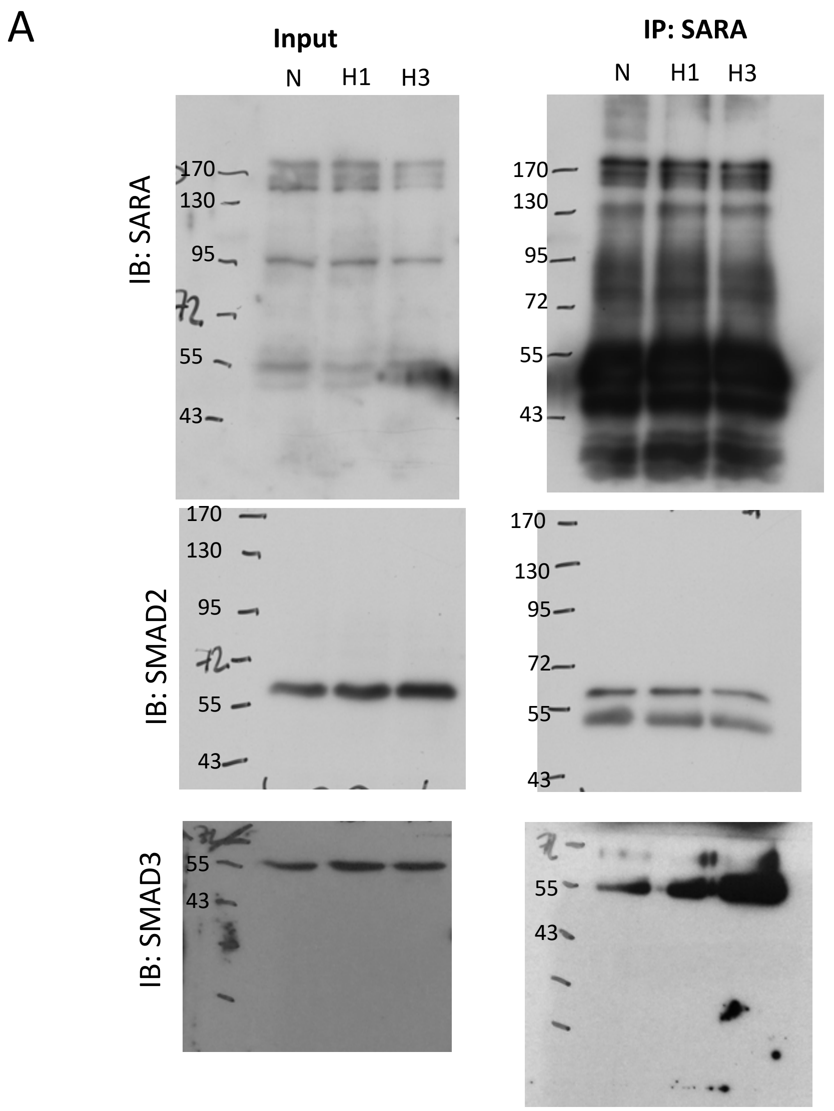

Figure S3 - Original blots

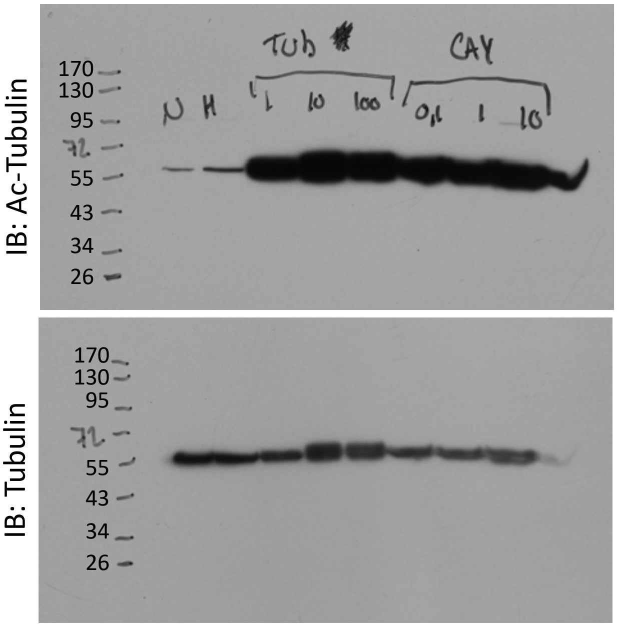

Figure S5 - Original blots

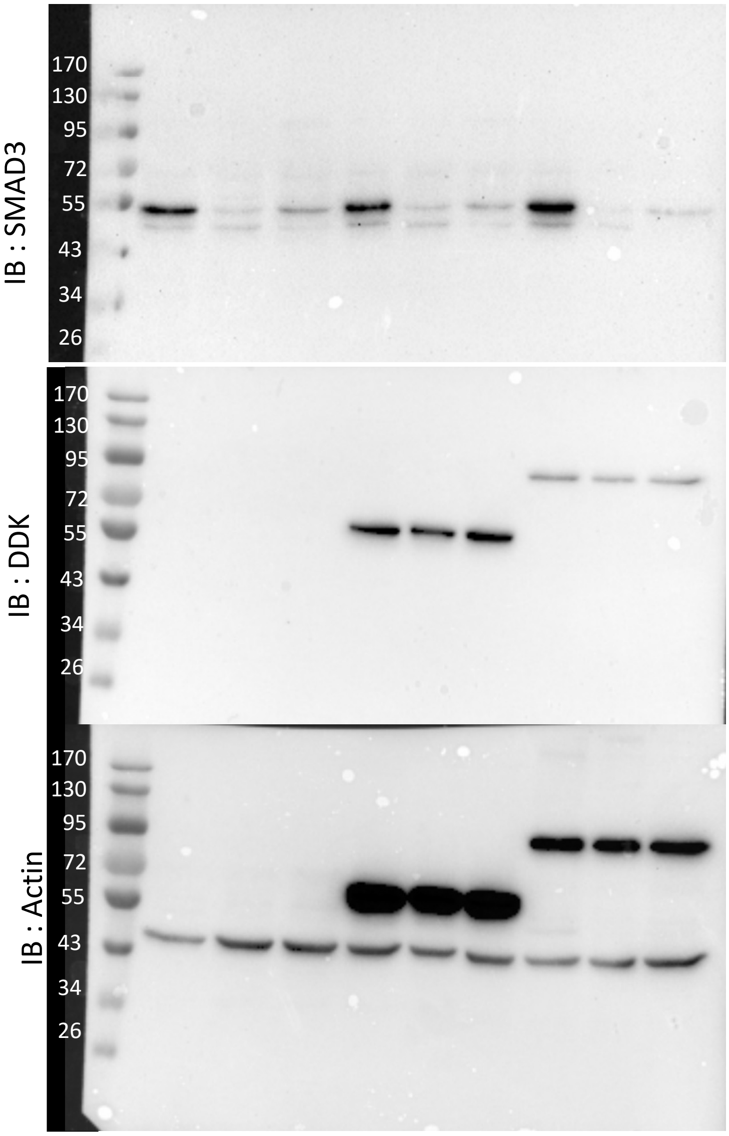

Supplement: Supplementary file 1 [file cancers-14-02751-s001.zip › cancers-1754862-supplementary/Supp Material/SuppFile1Proof.pdf]
